# A Phylogenetic and Functional Perspective on Volatile Organic Compound Production by *Actinobacteria*

**DOI:** 10.1128/mSystems.00295-18

**Published:** 2019-03-05

**Authors:** Mallory Choudoir, Sam Rossabi, Matthew Gebert, Detlev Helmig, Noah Fierer

**Affiliations:** a Cooperative Institute for Research in Environmental Sciences, University of Colorado, Boulder, Boulder, Colorado, USA; b Institute of Arctic and Alpine Research, University of Colorado, Boulder, Boulder, Colorado, USA; c Department of Ecology and Evolutionary Biology, University of Colorado, Boulder, Boulder, Colorado, USA; University of California, Irvine

**Keywords:** *Actinobacteria*, VOC, actinomycetes, microbial interactions, natural products, volatile organic compounds

## Abstract

Soil microbes produce an immense diversity of metabolites, including volatile organic compounds (VOCs), which can shape the structure and function of microbial communities. VOCs mediate a multitude of microbe-microbe interactions, including antagonism. Despite their importance, the diversity and functional relevance of most microbial volatiles remain uncharacterized. We assembled a taxonomically diverse collection of 48 *Actinobacteria* isolated from soil and airborne dust and surveyed the VOCs produced by these strains on two different medium types *in vitro* using gas chromatography-mass spectrometry (GC-MS). We detected 126 distinct VOCs and structurally identified approximately 20% of these compounds, which were predominately C_1_ to C_5_ hetero-VOCs, including (oxygenated) alcohols, ketones, esters, and nitrogen- and sulfur-containing compounds. Each strain produced a unique VOC profile. While the most common VOCs were likely by-products of primary metabolism, most of the VOCs were strain specific. We observed a strong taxonomic and phylogenetic signal for VOC profiles, suggesting their role in finer-scale patterns of ecological diversity. Finally, we investigated the functional potential of these VOCs by assessing their effects on growth rates of both pathogenic and nonpathogenic pseudomonad strains. We identified sets of VOCs that correlated with growth inhibition and stimulation, information that may facilitate the development of microbial VOC-based pathogen control strategies.

**IMPORTANCE** Soil microbes produce a diverse array of natural products, including volatile organic compounds (VOCs). Volatile compounds are important molecules in soil habitats, where they mediate interactions between bacteria, fungi, insects, plants, and animals. We measured the VOCs produced by a broad diversity of soil- and dust-dwelling *Actinobacteria in vitro*. We detected a total of 126 unique volatile compounds, and each strain produced a unique combination of VOCs. While some of the compounds were produced by many strains, most were strain specific. Importantly, VOC profiles were more similar between closely related strains, indicating that evolutionary and ecological processes generate predictable patterns of VOC production. Finally, we observed that actinobacterial VOCs had both stimulatory and inhibitory effects on the growth of bacteria that represent a plant-beneficial symbiont and a plant-pathogenic strain, information that may lead to the development of novel strategies for plant disease prevention.

## Abstract (precis)

Soil microbes produce a diverse array of natural products, including volatile organic compounds (VOCs). Volatile compounds are important molecules in soil habitats, where they mediate interactions between bacteria, fungi, insects, plants, and animals. We measured the VOCs produced by a broad diversity of soil- and dust-dwelling *Actinobacteria in vitro*. We detected a total of 126 unique volatile compounds, and each strain produced a unique combination of VOCs. While some of the compounds were produced by many strains, most were strain specific. Importantly, VOC profiles were more similar between closely related strains, indicating that evolutionary and ecological processes generate predictable patterns of VOC production. Finally, we observed that actinobacterial VOCs had both stimulatory and inhibitory effects on the growth of bacteria that represent a plant-beneficial symbiont and a plant-pathogenic strain, information that may lead to the development of novel strategies for plant disease prevention.

## INTRODUCTION

Microbial metabolism yields an extensive assortment of primary and secondary metabolites. While many microbial metabolites are nonvolatile, including many therapeutic antibiotics, microbes can also produce volatile organic compounds (VOCs) (microbial VOCs, or mVOCs). VOCs are small organic molecules (<C_20_) with low molecular masses (<300 Da) that are readily volatilized at ambient temperatures due to their high vapor pressures and low boiling points.

Many mVOCs are by-products of primary metabolism generated via aerobic heterotrophy, fermentation, amino acid catabolism, terpenoid biosynthesis, and sulfur reduction (1), while other mVOCs are produced via specialized secondary metabolic pathways (2). Some of the most commonly observed mVOCs are fatty acid derivatives (including alcohols, alkanes, and alkenes), aromatic compounds, nitrogen- and sulfur-containing compounds, and terpenoids (3, 4). To date, the chemical structures of approximately 2,000 VOCs from 1,000 bacterial and fungal species have been catalogued (5, 6). However, patterns of VOC production between closely related strains can vary considerably (7, 8), and many mVOCs remain uncharacterized, highlighting the vast potential for discovery and exploration of microbial volatiles.

Soil microbes are a particularly rich source of mVOCs (1, 9, 10). Under the unsaturated conditions typical of most soils, mVOCs can readily diffuse through air-filled pore spaces in the soil matrix (11), where they can influence rates of microbial activities associated with nitrogen and carbon transformations (12–14) and mediate biotic interactions between bacteria, fungi, plants, arthropods, insects, and animals (1, 15). mVOCs mediate microbe-microbe interactions in two major ways: by serving as infochemicals that influence morphology (16, 17), physiology, gene expression (18), and population dynamics (19) and by serving as agents of chemical warfare, competition, and antagonism, which can, in turn, shape the structure and function of soil communities (4).

Previous work has demonstrated that a broad collection of microbial volatiles can have both inhibitory and stimulatory growth effects on diverse pathogenic fungi (20–24). Furthermore, there is evidence that mVOCs may be the mode of action for pathogen control in disease-suppressive soils (25, 26), which underlines the practical applications of mVOCs as biocontrol agents. In fact, volatiles may represent the new frontier in antimicrobial product discovery (27). Despite their potential importance, the activities and functional relevance of most mVOCs remain unknown (28).

Soil-dwelling actinobacteria are an ideal group for studying mVOC production. Members of the phylum *Actinobacteria* are ubiquitous and abundant in soil habitats and are known to produce a wide range of secondary metabolites, including volatiles (7, 25, 29), with many of these biosynthetic pathways being evident from genomic analyses (30, 31). Actinomycetes are the predominant source of microbial-derived therapeutic antibiotics, antifungals, and other bioactive compounds (32, 33). The distribution of secondary metabolites between closely related actinomycetes reveals a strong correlation with phylogeny, suggesting that these compounds represent cohesive ecological traits (34–36). VOC analyses can resolve differences between *Streptomyces* species even better than commonly used marker gene sequences (25). However, it remains unclear if mVOCs produced by members of the phylum *Actinobacteria* are predictable from actinobacterial phylogeny. Resolving this knowledge gap is important for building a comprehensive understanding of mVOC production and for ultimately identifying how distinct lineages of bacteria differ with respect to their capacity to produce distinct VOCs.

Here, we assembled a culture collection of taxonomically diverse soil- and airborne dust-associated actinobacteria. We surveyed the diversity of VOCs produced by each of these strains *in vitro*. We then asked if more closely related strains had similar VOC emission profiles. Finally, we explored the functional potential of these VOCs by determining the effects of the actinobacterial VOCs on growth rates of both pathogenic and nonpathogenic pseudomonad test strains. Together, these results expand our understanding of actinobacterial VOC diversity and strengthen our knowledge of how microbial interactions can be mediated by mVOC production, information that could ultimately inform development of strategies to control soilborne pathogens.

## RESULTS

### Actinobacterial volatile organic compound emission profiles.

**FIG 1:**
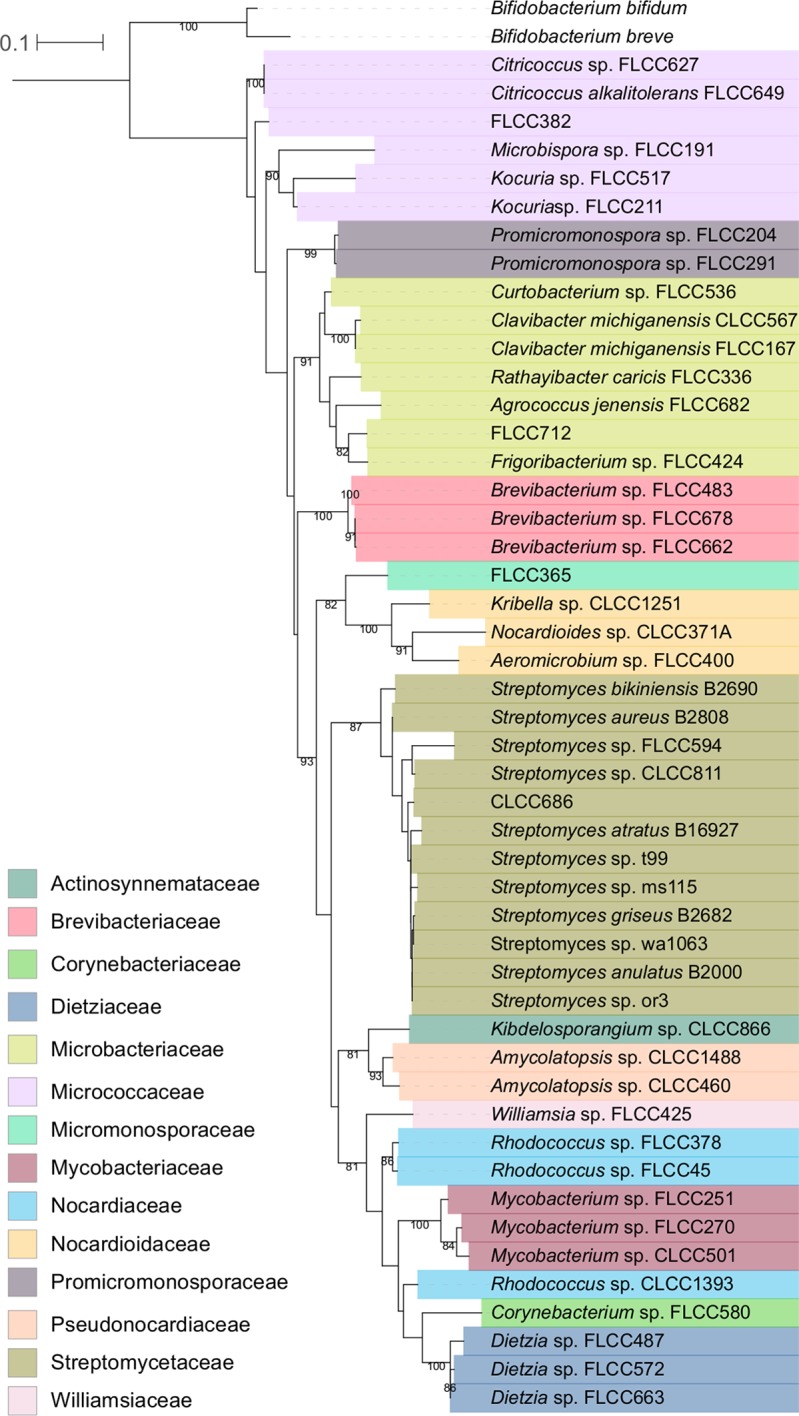
Tree depicting the phylogenetic relationships of all strains included in this study. We surveyed VOCs produced by 48 soil- and airborne-dust-associated actinobacterial strains. The phylogeny was constructed from nucleotide alignments of partial 16S rRNA gene sequences using maximum likelihood and a GTRGAMMA model of evolution. The bar indicates nucleotide substitutions per site. Nodes with bootstrap support values of ≥70 are labeled. The tree was rooted with Bifidobacterium bifidum strain JCM 1255 and Bifidobacterium breve strain KSS01. Strain names reflect their isolation conditions and culture collection of origin (see Table S1 in the supplemental material). Strains are colored by their taxonomic assignment at the family level according to the key, and taxonomic assignments at the genus and species levels are included when available. The family *Nocardiaceae* is not monophyletic.

We assembled a set of 48 soil- and airborne dust-associated actinobacterial strains that represented 14 taxonomic families within the phylum *Actinobacteria* (Fig. 1; see also Table S1 in the supplemental material), and this collection well represents the overall taxonomic diversity within the phylum (37). We used a gas chromatography-mass spectrometry (GC-MS) method to survey the chemical diversity of VOCs produced by these strains grown on different medium types, glycerol arginine (GA) agar, a minimal sporulation medium, and International *Streptomyces* Project Medium 2 (ISP2) agar, a complex rich medium containing yeast and malt extracts (Table S2), as previous studies have shown that medium type influences the types of VOCs produced by microbes *in vitro* (38, 39). Not all strains grew well on both medium types, so we measured VOC production for 46 and 34 strains representing 14 and 11 taxonomic families on ISP2 and GA media, respectively, with the GA data set omitting the families *Brevibacteriaceae*, *Corynebacteriaceae*, and *Dietziaceae* (Fig. S1).

10.1128/mSystems.00295-18.1 TABLE S1 Strain name, taxonomic classification, culture collection, isolation information, and NCBI accession numbers (when available) for the 48 actinobacterial strains used in this study. 16S rRNA gene sequences are located in Data Set S1 in the supplemental material. The 24 strains used for the pseudomonad growth assay are indicated with asterisks. Originating culture collections include the Agricultural Research Service (NRRL) (Peoria, IL, USA) culture collection (https://nrrl.ncaur.usda.gov/), the Fierer Lab culture collection (University of Colorado, Boulder, Boulder, CO, USA), and the Buckley Lab culture collection (Cornell University, Ithaca, NY, USA). See Table S2 in the supplemental material for medium recipes. Fierer Lab strains were isolated from surface soils (0 to 5 cm) sampled from a subalpine forest in Boulder County (Boulder, CO, USA) and from airborne dust samples from glass bead traps installed at the Boulder Atmospheric Observatory (BAO) research tower (Erie, CO, USA) for 2 weeks during June 2016. Buckley Lab strains were isolated from grassland soils across the United States (see references 54 and 55). Download Table S1, DOCX file, 0.1 MB. Copyright © 2019 Choudoir et al. 2019 Choudoir et al. This content is distributed under the terms of the Creative Commons Attribution 4.0 International license.

10.1128/mSystems.00295-18.2 TABLE S2 Medium recipes used for actinobacterial strain isolation, VOC surveys, and pseudomonad growth assays. Download Table S2, DOCX file, 0.1 MB. Copyright © 2019 Choudoir et al. 2019 Choudoir et al. This content is distributed under the terms of the Creative Commons Attribution 4.0 International license.

10.1128/mSystems.00295-18.5 FIG S1 The number of VOCs produced varies between actinobacterial strains. Bars show the total number of distinct VOCs detected (a) and the total number of distinct VOCs detected on GA and ISP2 media (b) for each strain. Strains unable to grow on GA or ISP2 medium are marked with asterisks (b). Download FIG S1, EPS file, 0.1 MB. Copyright © 2019 Choudoir et al. 2019 Choudoir et al. This content is distributed under the terms of the Creative Commons Attribution 4.0 International license.

**FIG 2:**
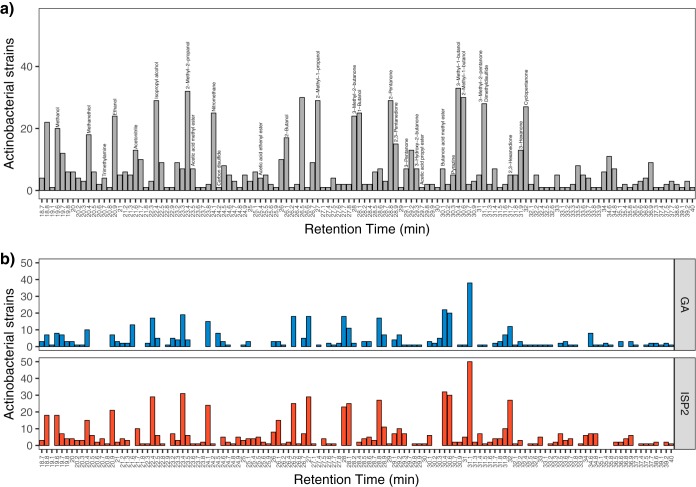
Identification of a total of 126 VOCs (see Table S3 in the supplemental material) produced by 48 actinobacterial strains. Distinct volatiles are ordered by their retention times and approximately by increasing molecule size. Bars show the total number of actinobacterial strains that produced each VOC (a) and the total number of strains that produced each VOC on GA and ISP2 media (b). The 28 compounds identified from their mass spectra are labeled (a) (see Materials and Methods and Table S4). Note that two identified compounds coeluted at the same retention time of 31.1 min, 3-methyl-2-pentanone and dimethyl disulfide.

We detected a total of 126 distinct VOCs across all samples (Fig. 2a), with 92 and 108 compounds detected on GA and ISP2 media, respectively (Fig. 2b). Seventy-four compounds were detected on both medium types, while 34 VOCs were ISP2 specific and 18 VOCs were GA specific (Fig. 2b). Of these, we were able to verify the chemical structures of 28 compounds based on their mass spectra (see Materials and Methods) (Fig. 2a and Table S4). A total of 31% of these compounds were alcohols, 31% were ketones, and the remaining VOCs were esters or nitrogen- or sulfur-containing compounds (Table 1). We identified over 90% of the most abundant VOCs (i.e., VOCs detected in ≥24 samples), and these included 3-methyl-1-butanol (*n* = 33 samples), 2-methyl-2-propanol (*n* = 32 samples), 2-methyl-1-butanol (*n* = 30 samples), 2-methyl-1-propanol and 2-pentanone (*n* = 29 samples), and 3-methyl-2-pentanone/dimethyl disulfide (DMDS) (*n* = 28 samples) (Fig. 2a). Conversely, over 77% of the volatile compounds detected could not be identified, mostly because of the low signal intensity in their mass spectra. Many of these unidentified compounds were detected in few samples and produced by only a subset of the actinobacterial strains (Fig. 2).

**TABLE 1:** Identification of 28 compounds that can be categorized into five broad chemical classes^*a*^

Class	Compounds
Alcohols	Methanol, ethanol, isopropyl alcohol, 2-methyl-2-propanol, 2-methyl-1-propanol, 1-butanol, 2-butanol, 3-methyl-1-butanol, 2-methyl-1-butanol
Esters	Acetic acid methyl ester, acetic acid ethenyl ester, acetic acid propyl ester, butanoic acid methyl ester
Ketones	3-Methyl-2-butanone, 2-pentanone, 3-pentanone, 2,3-pentanedione, 3-methyl-2-pentanone, 3-hydroxy-2-butanone, 2,3-hexanedione, 3-hexanone, cyclo-pentanone
Nitrogen-containing compounds	Trimethylamine, nitromethane, pyrazine*, acetonitrile*
Sulfur-containing compounds	Methanethiol, carbon disulfide, dimethyl disulfide

a Of the 126 distinct VOCs detected, we were able to identify the chemical structures of 28 compounds, which can be categorized into five broad chemical classes. Asterisks indicate compounds detected in the ISP2 medium blanks but not the GA medium blanks.

10.1128/mSystems.00295-18.3 TABLE S3 Identification of a total of 126 VOCs produced by 48 actinobacterial strains. Shown is the presence (1) or absence (0) of distinct VOCs ordered by retention index and retention time (minutes) for each strain grown on GA and ISP2 medium types. Rows with “NA” indicate the 18 and 2 strains that did not grow on GA and ISP2 medium types, respectively. Total VOCs are the number of compounds identified as distinct peaks in FID chromatograms. Columns highlighted in gray denote peaks identified by their mass spectra (see Table S4 in the supplemental material). Asterisks indicate compounds detected in the ISP2 medium blanks but not the GA medium blanks, and these compounds could represent sterile medium emissions. Download Table S3, PDF file, 0.4 MB. Copyright © 2019 Choudoir et al. 2019 Choudoir et al. This content is distributed under the terms of the Creative Commons Attribution 4.0 International license.

10.1128/mSystems.00295-18.4 TABLE S4 Of the 126 distinct VOCs detected, the chemical structures of 28 compounds were identified from their mass spectra. For each compound, retention time (minutes), retention index, compound identity, mass spectra (*m/z* relative abundance), mass spectra for the top match from the NIST mass spectral search program, and percent library match are reported. Note that two identified compounds coeluted at the same retention time of 31.1 min, 3-methyl-2-pentanone and dimethyl disulfide. Asterisks indicate compounds detected in the ISP2 medium blanks but not the GA medium blanks, and these compounds could represent sterile medium emissions. Download Table S4, DOCX file, 0.1 MB. Copyright © 2019 Choudoir et al. 2019 Choudoir et al. This content is distributed under the terms of the Creative Commons Attribution 4.0 International license.

Of note, we did not identify geosmin or 2-methylisobororneol in our mVOC collection, but these compounds are often observed to be produced by actinomycetes and contribute to the distinct “earthy” or “musty” smell of soil (7, 40). It is possible that one of the unknown compounds is in fact geosmin or that, under the sampling conditions used here, geosmin and other volatiles that were previously detected from actinobacteria were not produced in sufficient quantities to be detected. Importantly, our abilities to detect and identify the VOCs are limited by the specific analytical methods used here. The methods were not able to detect all types of VOCs, such as methane or highly polar VOCs with multifunctional groups. Thus, the detected VOCs likely represented an undetermined fraction of the total VOCs emitted from these samples.

**FIG 3:**
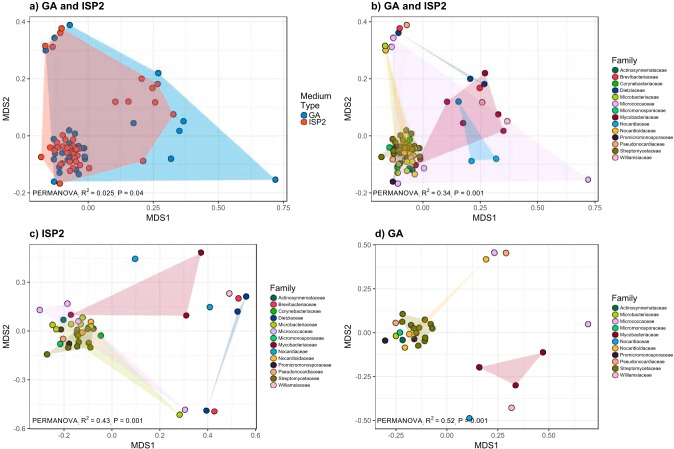
Nonmetric multidimensional scaling (NMDS) illustrates the differentiation of actinobacterial VOC emission profiles. Each point depicts the Jaccard distances between VOC profiles of strains grown on GA and ISP2 media (a and b) and on ISP2 medium (c) and GA medium (d) alone. Strains that did not grow on a given medium type and strains that produced no detectable VOCs were removed to minimize distance in the matrix. Points are colored to reflect the variation explained by medium type (a) and by taxonomic assignment at the family level (b to d) according to the key.

**FIG 4:**
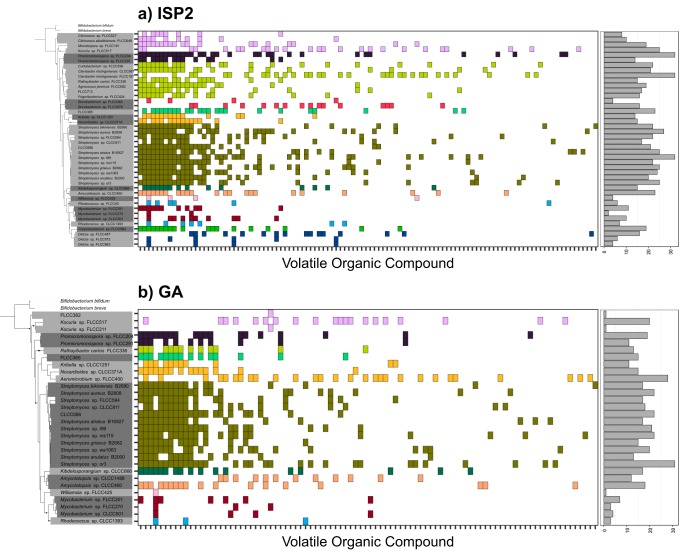
Closely related actinobacterial strains produced similar VOC profiles. In the left panels, trees reflect the phylogenetic relationships of 16S rRNA gene sequences between actinobacterial strains (Fig. 1). The 6 and 18 strains that did not grow or produced no detectable mVOCs on ISP2 and GA medium types, respectively, were trimmed from the phylogeny. Deleted leaves and nodes in the resulting tree are indicated by black circles. In the center panels, the colored boxes along the *x* axis depict distinct volatiles produced by strains grown on ISP2 (a) and GA (b) medium types. Volatiles are ordered by the number of strains that produced each compound. Boxes of the same color are VOCs produced by strains sharing the same taxonomic assignment at the family level. In the right panels, bars show the total number of VOCs produced by each strain on each medium type.

We detected 0 to 36 total distinct VOCs per strain, and in general, the number of VOCs produced differed between medium types (Fig. S1 and Table S3). Two strains, FLCC378 and FLCC662, produced no VOCs above our level of detection or what was emitted from the sterile medium blanks. Most VOCs were produced by very few strains such that 24.6% of total VOCs were strain specific, and only 11 VOCs were produced by more than 50% of strains (Fig. S2a). This right-skewed frequency distribution was consistently observed for VOCs produced on GA and ISP2 media (Fig. S2b). Each actinobacterial strain produced a unique combination of VOCs (Fig. 3 and 4 and Table S3). Actinobacterial VOC profiles differed between ISP2 and GA medium samples (*R*^2^ = 0.025; *P = *0.04 [by permutational multivariate analysis of variance {PERMANOVA}]) (Fig. 3a), indicating that strains produced different VOCs when grown on different medium types. Despite this effect of medium type on VOC profiles, strain-level differences explained far more of the variation in VOC profiles (*R*^2^ = 0.82; *P = *0.001 [by PERMANOVA]) (Fig. 3b). Namely, the strain-level variation in VOC profiles exceeded the variation in VOC profiles observed across the two medium types.

10.1128/mSystems.00295-18.6 FIG S2 Most VOCs were produced by few actinobacterial strains, and few VOCs were produced by many strains. The histogram depicts the frequency distribution of distinct VOCs produced by actinobacterial strains (a) and of VOCs produced on GA and ISP2 medium types (b). Strains that did not grow on a given medium type and strains that produced no detectable VOCs were removed. Download FIG S2, EPS file, 0.2 MB. Copyright © 2019 Choudoir et al. 2019 Choudoir et al. This content is distributed under the terms of the Creative Commons Attribution 4.0 International license.

### Taxonomic and phylogenetic signals of volatile organic compound production.

Next, we determined if the strain-level variations in actinobacterial VOC emissions were predictable from taxonomic or phylogenetic differences between the strains. First, we asked if the number of distinct VOCs varied between strains and across taxonomic groups. We observed a taxonomic signal in the number of unique VOCs produced, and the number of total VOCs per strain varied across the taxonomic families (*F*_13,34_ = 5.7; *P < *0.001 [by analysis of variance {ANOVA}]). For example, strains within the family *Streptomycetaceae* produced more distinct VOCs per strain than the families *Brevibacteriaceae*, *Dietziaceae*, *Micrococcaceae*, *Mycobacteriaceae*, and *Nocardiaceae* (*P < *0.01 [by Tukey’s *post hoc* test]).

Next, we asked if VOC emission profiles varied depending on the taxonomic identity of the strains or their phylogenetic relationships. In other words, did more closely related strains emit more similar types of VOCs? Strain-level VOC profiles varied depending on taxonomy at the family-level classification (*R*^2^ = 0.34; *P = *0.001 [by PERMANOVA]), and this variation was consistently observed for strains grown on GA medium (*R*^2^ = 0.52; *P = *0.001 [by PERMANOVA]) and on ISP2 medium (*R*^2^ = 0.43; *P = *0.002 [by PERMANOVA]) (Fig. 3b to d). For example, strains FLCC204 and FLCC291 within the family *Promicromonosporaceae* consistently emitted an unknown VOC (retention time of 32.5 min) across both medium types, and this VOC was not emitted from other actinobacterial strains outside this family. Hence, there were specific VOCs that were emitted exclusively across strains within the same taxonomic group (Fig. 4). We also observed a phylogenetic signal in VOC production such that strains that were more genetically similar (estimated from the similarity in their 16S rRNA gene sequences) also had more similar VOC profiles on both ISP2 (Mantel *r* = 0.22; *P = *0.005) and GA (Mantel *r* = 0.37; *P = *0.001) media (Fig. S3).

10.1128/mSystems.00295-18.7 FIG S3 Actinobacterial VOC profiles are more different with increasing genetic distance. Each point depicts the pairwise comparisons between VOC profile Jaccard dissimilarities and 16S rRNA gene nucleotide distances for compounds detected on ISP2 (a) and GA (b) medium types. Strains that did not grow on a given medium type and strains that produced no detectable VOCs were removed to minimize distance in the matrix. Blue lines depict the linear regression, with gray shading indicating 95% confidence intervals. Download FIG S3, EPS file, 2 MB. Copyright © 2019 Choudoir et al. 2019 Choudoir et al. This content is distributed under the terms of the Creative Commons Attribution 4.0 International license.

Despite overall strong taxonomic and phylogenetic signals, an appreciable number of VOCs were produced by distantly related strains. For instance, 2-methyl-2-propanol was detected in at least one strain representing 13 of the 14 taxonomic families, and all families shared at least one VOC with strains from a different family (Fig. 4). For example, while the family *Streptomycetaceae* had the largest collection of family-specific VOCs (21 unique VOCs on ISP2 medium and 24 unique VOCs on GA medium), strains within this family also shared 19 and 38 VOCs across taxonomic groups on ISP2 and GA media, respectively (Fig. 4).

### Effect of volatiles on pseudomonad growth.

Finally, we asked if volatiles produced by actinobacteria influenced the growth of two pseudomonad test strains: Pseudomonas fluorescens SBW25, a plant growth-promoting symbiont (41), and Pseudomonas syringae pv. tomato DC300, the causative agent of bacterial speck of tomato (42). To answer this question, we designed an assay that exposed pseudomonad test strains throughout the course of a growth curve to the volatiles emitted by 24 actinobacterial strains and measured differences in growth rates compared to growth rates of the pseudomonads exposed to sterile medium blanks. Briefly, both actinobacterial and pseudomonad strains were grown in adjacent wells of a culture plate, which allowed VOCs to diffuse across the shared headspace, and pseudomonad growth was measured continuously on a plate reader (see Materials and Methods and Fig. S4 in the supplemental material).

10.1128/mSystems.00295-18.8 FIG S4 Nonpathogenic Pseudomonas fluorescens SBW25 (SBW25) and pathogenic Pseudomonas syringae pv. tomato DC3000 (DC3000) were exposed to VOCs produced by actinobacterial strains throughout exponential growth (see Materials and Methods). (a) Briefly, actinobacterial cell suspensions were plated onto ISP2 agar on alternating rows of a 96-well culture plate and incubated for 5 days at 30°C. Cultures of pseudomonad test strains grown overnight were diluted into fresh King’s B medium (OD at 600 nm = 0.05) and transferred into culture plates. (b) Thus, the pseudomonad cultures grown overnight shared a headspace with actinobacterial strain cultures grown for 5 days, allowing mVOCs to diffuse across the plate. The culture plate was incubated at 24°C with shaking on a plate reader. (c) The absorbance at 630 nm was measured every 20 min for a total of 980 min, and the absorbance values of medium blanks were subtracted from each measurement. Download FIG S4, EPS file, 2.3 MB. Copyright © 2019 Choudoir et al. 2019 Choudoir et al. This content is distributed under the terms of the Creative Commons Attribution 4.0 International license.

**FIG 5:**
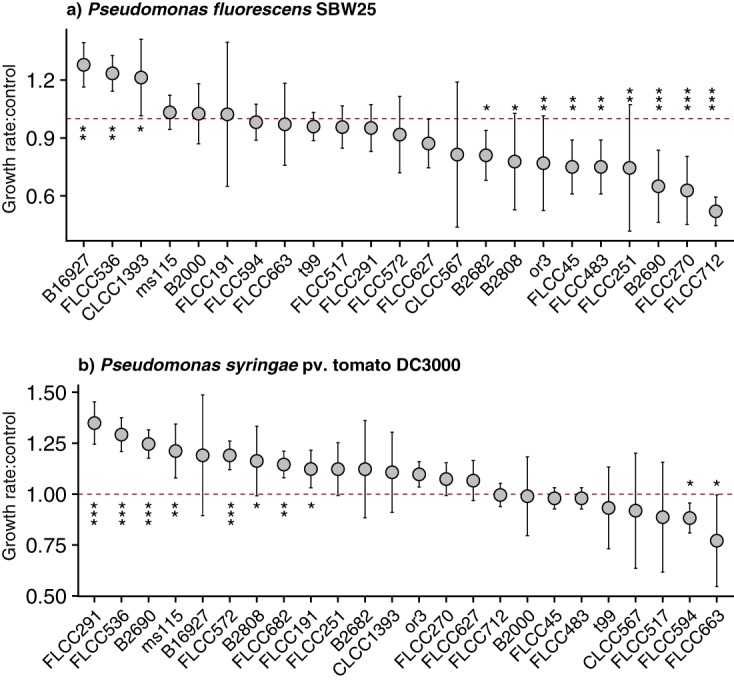
Actinobacterial VOCs correlate with effects on growth rates of pseudomonad test strains. Nonpathogenic Pseudomonas fluorescens SBW25 (a) and pathogenic Pseudomonas syringae pv. tomato DC3000 (b) were exposed to actinobacterial VOCs throughout exponential growth (see Materials and Methods and Fig. S4 in the supplemental material). Circles show the mean ratios of pseudomonad growth rates in the presence of actinobacterial VOCs (strain names are included on the *x* axis) to pseudomonad growth rates in the presence of sterile medium blanks (i.e., control), and error bars show the standard deviations. If the ratio of the growth rate to the control growth rate, or “Growth rate:control,” equals 1 (i.e., dashed red line), this indicates no difference in the growth rate compared to that of the control; values of >1 indicate growth stimulation, and values of <1 indicate growth inhibition. Pseudomonad growth rates that were significantly different from those of the controls are marked with asterisks (*, *P* < 0.05; **, *P* < 0.01; ***, *P* < 0.001 [by a *t* test without *P* value adjustment]).

Actinobacterial volatiles correlated with both stimulatory and inhibitory effects on pseudomonad growth rates. For P. fluorescens SBW25, nine strains were associated with a significant decrease in the growth rate, while three strains were associated with a significant increase in the growth rate (Fig. 5a). For P. syringae pv. tomato DC3000, two strains were associated with a significant decrease in the growth rate, while eight strains were associated with a significant increase in the growth rate (Fig. 5b). In some cases, the magnitude of the growth effects was quite large. For example, exposure to the volatiles of strain FLCC712 correlated with a 52% reduction in the growth of P. fluorescens SBW25 compared to that of the medium blank. Conversely, exposure to the VOCs of strain FLCC291 correlated with a growth rate for P. syringae pv. tomato DC3000 that was 135% higher than that of the medium blank.

**FIG 6:**
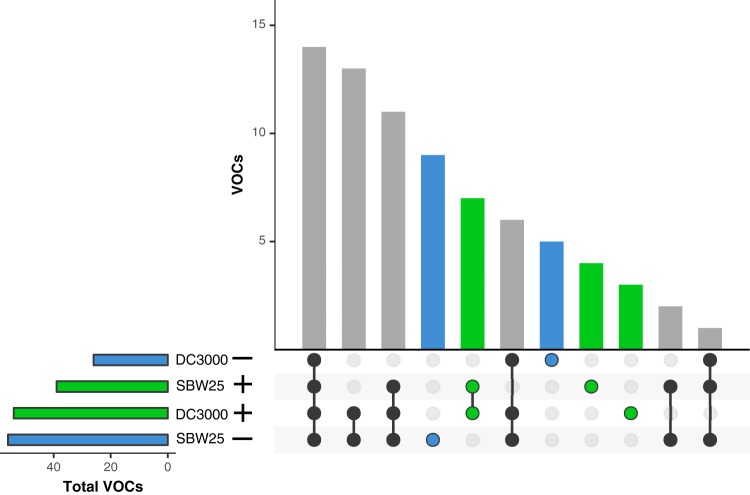
Sets of actinobacterial VOCs are associated with effects on growth rates of pseudomonad test strains. We restricted this analysis to strains that were correlated with significant differences in growth rates compared to controls. The left panel shows the total number of distinct VOCs from all actinobacterial strains associated with growth inhibition (blue) (−) or stimulation (green) (+) of nonpathogenic Pseudomonas fluorescens SBW25 (SBW25) and pathogenic Pseudomonas syringae pv. tomato DC3000 (DC3000). The bars and matrix in the right panel show the collections of discrete VOCs that were unique to pseudomonad strain specific growth stimulation or inhibition. Blue circles depict collections of VOCs that were exclusive to growth inhibition, and green circles depict collections of VOCs that were exclusive to growth stimulation. Black circles depict collections of VOCs that were associated with both growth stimulation and inhibition.

We were able to identify discrete actinobacterial VOCs that were associated with inhibitory or stimulatory growth effects on pseudomonads (Fig. 6). Fifty-six and 39 compounds comprised the total collection of VOCs produced by actinobacterial strains that were associated with inhibited and stimulated growth, respectively, of P. fluorescens SB525. Twenty-six and 54 compounds comprised the total collection of VOCs that were associated with inhibited and stimulated growth, respectively, of P. syringae pv. tomato DC3000. There were nine and five discrete VOCs that were exclusively correlated with growth rate inhibition of P. fluorescens SB525 and P. syringae pv. tomato DC3000, respectively (Fig. 6). For instance, butanoic acid methyl ester was associated with inhibition of P. syringae pv. tomato DC3000. There were four and three discrete VOCs that were exclusively correlated with growth rate stimulation of P. fluorescens SB525 and P. syringae pv. tomato DC3000, respectively, and a shared set of seven discrete VOCs that correlated with stimulated growth of both pseudomonads, including 3-hydroxy-2-butanone (Fig. 6). However, besides the two VOCs mentioned above, the chemical identities of these compounds associated with significant differences in pseudomonad growth rates could not be determined.

## DISCUSSION

Soil microbes are a rich source of metabolites, including some that are mVOCs, which have long been of interest given their potential to serve as carbon and nutrient sources (43, 44) or to act as infochemicals that mediate a multitude of biotic interactions (1, 15). Despite a large catalogue of mVOCs (5, 6), the majority of mVOCs remain uncharacterized in terms of both their chemical structure and activity. Furthermore, we lack a comprehensive understanding of the evolutionary and ecological processes that generate and maintain this vast mVOC diversity in soil ecosystems. Given these knowledge gaps, we used GC-MS to understand the diversity of VOCs produced by soil- and dust-associated bacteria within the phylum *Actinobacteria* (Fig. 1), viewed through a phylogenetic and functional lens.

The VOCs detected here ranged in carbon content from about C_3_ to C_10_. We were able to structurally identify 22% of the 126 distinct compounds, which spanned broad chemical classes, including alcohols, esters, ketones, and nitrogen- or sulfur-containing compounds (Table 1; see also Table S4 in the supplemental material). Previous studies have also detected diverse collections of mVOCs, and in particular, the types of volatiles detected here are routinely identified in surveys of microbial soil communities and of soil-derived microbial isolates (see references 1 and 10 and references therein). Similar to our study, previous mVOC studies using GC-MS methods also reported a high percentage of unknowns (9, 22, 45), suggesting a need for improved analytical capabilities for identifying polar and multifunctional volatiles.

Many of the abundant compounds identified here are likely the by-products of primary metabolic pathways, which are generally conserved at the phylum level. For example, the alcohols 3-methyl-1-butanol, 2-methyl-1-butanol, 2-methyl-1-propanol, and 1-butanol were some of the most abundant VOCs detected here (Fig. 2 and Table 1), and these were also among the most abundant compounds detected in other actinomycetes (7). Alcohols are often generated through amino acid metabolism (4), and 3-methyl-1-butanol is a known by-product of leucine catabolism (46). We also identified a number of branched ketones, including 3-methyl-2-butanone, 3-pentanone, and 3-methyl-2-pentanone, and cyclo-pentanone (Fig. 2 and Table 1), which are characteristic of actinomycetes (47). In addition, dimethyl disulfide (DMDS) is commonly produced by diverse actinobacterial species (Fig. 2 and Table 1) (47). DMDS has been shown to display antimicrobial and pesticidal properties (2) and to also inhibit quorum sensing (19).

Each strain produced a unique VOC profile (Fig. 3 and 4 and Table S3). While strain-level differences explained most of the variation in VOC profiles, we also observed a medium effect (Fig. 3 and Fig. S1), and this is consistent with previous studies that have also shown that differences in growth media can influence the types of VOCs produced by individual strains *in vitro* (38, 39). Indeed, there are various factors that may influence the types and amounts of VOCs produced, including, but not limited to, nutrient conditions, underlying genomic variation, microbial growth phase and cell morphology, and proximity to other species. While all strains were surveyed for VOC production at 12 to 13 days of incubation on solid media, not all strains were necessarily at the same growth stage at this point, thus potentially contributing to unmeasured variation in VOC profiles. Consequently, the VOCs identified here likely represent a fraction of a broader chemical potential of these isolates, and we would expect to detect different VOCs under various growth conditions.

We observed a taxonomic and phylogenetic signal for mVOCs such that more closely related actinobacterial strains had more similar VOC emission profiles (Fig. 3 and 4 and Fig. S3). As discussed above, many of the abundant mVOCs are likely by-products of central metabolism and thus represent evolutionarily cohesive traits conserved across broader taxonomic scales. However, most of the VOCs were strain specific (Fig. S2), a pattern that was previously observed for mVOCs (22) and for secondary metabolite biosynthetic gene clusters (48). Of note, *Streptomyces* produces, on average, the highest number of distinct VOCs per strain (22 to 34 VOCs) (Fig. 4), and this aligns with other studies that identified 14 to 42 VOCs emitted per streptomycete (7). Within the phylum *Actinobacteria*, the genus *Streptomyces* is known for its prolific production of secondary metabolites and natural products (49), and correspondingly, it is unsurprising that *Streptomyces* also produced the most unique VOC profiles here. These results suggest that mVOCs can act as dynamic agents of evolutionary and ecological processes shaping finer scales of diversity (34–36).

Given the chemical diversity of actinobacterial mVOCs detected in our survey, we propose that these compounds also possessed a broad functional diversity. Furthermore, studies suggest that actinobacteria, and *Streptomyces* species in particular, are important keystone taxa in disease-suppressive soils (26, 50, 51). We found that actinobacterial VOCs correlated with both stimulatory and inhibitory effects on the growth rates of plant growth-promoting and phytopathogenic pseudomonad test strains, and in some instances, these effects were quite large (Fig. 5). While we focused on the maximum growth rate, it is also possible that actinobacterial volatiles could impact additional pseudomonad growth characteristics, such as increased lag time to exponential phase. In addition, it is possible that the pseudomonad strains also emitted mVOCs, which in turn resulted in the production of different actinobacterial VOCs.

We identified a collection of nine and five mVOCs that were exclusively associated with growth inhibition of P. fluorescens SBW25 and P. syringae pv. tomato DC300, respectively (Fig. 6). There was not a strong phylogenetic signal for growth suppression or enhancement for either pseudomonad strain, although closely related strains occasionally had similar phenotypes (e.g., *Streptomyces* strains B2690 and B2808 were both associated with growth inhibition of the beneficial pseudomonad and growth stimulation of the pathogenic pseudomonad). Of particular interest, butanoic acid methyl ester correlated with growth inhibition of the phytopathogenic pseudomonad, and this compound has been shown to exhibit antimicrobial activity against other microbial pathogens (52, 53). Future work should focus on confirming the specific activity and identity of these actinobacterial VOCs, either individual compounds or combinations of compounds, that were associated with altered pseudomonad growth rates.

This study highlights the importance of viewing mVOC diversity within a phylogenetic framework and shows that actinobacteria can produce a vast repertoire of uncharacterized microbial natural products important in mediating microbe-microbe interactions. In particular, some VOCs were associated with growth inhibition of pseudomonad strains that have previously been shown to be important to plant health. More broadly, this work highlights the potential utility of leveraging mVOC-based solutions for pathogen control in agricultural ecosystems.

## MATERIALS AND METHODS

### Actinobacterial strain isolation.

The actinobacterial strains used in this study were obtained from various culture collections (see Table S1 in the supplemental material). *Streptomyces* sp. strain ms115, *Streptomyces* sp. strain or3, *Streptomyces* sp. strain t99, and *Streptomyces* sp. strain wa1063 were isolated from grassland soils across the United States, as previously described (54, 55). Type strains Streptomyces anulatus B2000, Streptomyces atratus B16727, Streptomyces aureus B2808, Streptomyces bikiniensis B2690, and Streptomyces griseus B2682 were obtained from the Agricultural Research Service (NRRL) culture collection (https://nrrl.ncaur.usda.gov/). The remaining strains were isolated at the University of Colorado, Boulder, between 2016 and 2017. Briefly, soil and airborne dust samples were plated onto solid media and incubated at 25°C for approximately 3 weeks. Actinobacterial colonies were transferred from the enrichment plates into Axygen 2-ml 96-deep-well plates (Corning Life Sciences, Tewksbury, MA, USA) containing liquid medium and incubated at 25°C for 6 to 9 weeks. Strains were streaked for final isolation on solid medium (see Table S1 for additional isolation information and Table S2 for medium recipes). Prior to the experiment, strain purity was verified through multiple rounds of streaking for isolation on ISP2 agar.

### 16S rRNA gene sequencing and phylogenetic analyses.

Full-length 16S rRNA gene sequences were obtained from public sequence databases when available. For all remaining strains, small-subunit (SSU) rRNA gene sequences were amplified with primers 27F (GTGCTGCAGAGAGTTTGATCCTGGCTCAG) and 1492R (CACGGATCCTACGGGTACCTTGTTACGACTT) (56) with the following 24-µl PCR mixture: 12.5 µl GoTaq Hot Start master mix (Promega, Madison, WI, USA), 10.5 µl H_2_O, 0.5 µl forward primer from a 10 mM stock, 0.5 µl reverse primer from a 10 mM stock, and a direct-from-colony template. The following thermocycler conditions were used: 98°C for 10 min and 35 cycles of 94°C for 1 min, 55°C for 1 min, 72°C for 2 min, and 72°C for 10 min, followed by a short-term hold at 4°C. Genewiz (South Plainfield, NJ, USA) generated Sanger sequences from the amplicon using sequencing primer 27F. Automatic base calling and quality control of trace files were performed using Phred (57). Taxonomy was determined using the Ribosomal Database Project classifier (57, 58) trained on the Greengenes 13_8 16S rRNA database (59). See the supplemental material for the actinobacterial 16S rRNA gene sequences.

Phylogenetic relationships were determined from partial 16S rRNA gene sequences. 16S rRNA sequences were aligned using MAFFT (60), and poorly aligned regions were removed with trimAL (61), resulting in an aligned nucleotide fragment of 682 bp. A maximum likelihood (ML) tree was constructed using the generalized time-reversible nucleotide substitution model (62) with gamma-distributed rate heterogeneity among sites (GTRGAMMA) in RAxML v7.3.0 (63). Bootstrap support was determined from 100 inferences using the RAxML rapid bootstrapping algorithm (64). The tree was rooted with Bifidobacterium bifidum JCM 1255 and Bifidobacterium breve KSS01.

### Volatile organic compound collection and sampling.

Volatile organic compounds (VOCs) produced by the actinobacterial strains were characterized using a dynamic flux chamber system with a custom sampling manifold and a VOC preconcentration inlet system (65). VOC profiles were quantified only for strains that grew readily on a given medium type (46 strains on ISP2 medium and 30 strains on GA medium) (Fig. S1 and Table S3). Strains were plated onto triplicate 60-mm petri plates with both ISP2 and GA agar medium types and incubated at 30°C for 12 to 13 days, which resulted in a heavy lawn of growth coating the entire plate. Triplicate plates were placed in 475-ml glass jars and maintained at 23°C throughout VOC sampling, as described below.

For VOC analyses, the jars containing the culture plates were connected to a dynamic flowthrough system with zero air flowed through the jars at 140 ml min^−1^. Zero air was generated by passing compressed air through a custom zero air generator, which oxidizes hydrocarbons to CO_2_. Samples were collected after culture plates had been exposed to the zero air flow for between 10 min and 8 h. Of note, there may be a dependence of the intensity of VOC emission peaks on the flushing time. Because of this uncertainty, results are presented in a solely qualitative manner.

For VOC analyses, a fraction of the exhaust purge flow was collected at a rate of 50 ml min^−1^ for 40 min, resulting in a 2-liter sample volume. Samples were first drawn through a Peltier-cooled (−20°C) trap to remove water vapor by freezeout from the sample stream. Next, VOCs were collected on a cooled (−30°C) microadsorbent trap that contained 25 mg of Carboxen 1016 and 220 mg of Carboxen 1000 solid adsorbents (Sigma-Aldrich, St. Louis, MO, USA). After sample collection, VOCs were mobilized by rapidly heating the trap to 290°C for injection onto the gas chromatography (GC) column. A gas chromatograph (Hewlett Packard 5890)/flame ionization detector/mass spectrometer (Agilent 5971) (GC/FID/MS) instrument (Agilent Technologies, Boulder, CO, USA) was used. Separation was achieved with a 0.32-mm-internal-diameter (ID) by 60-m-long, 5-µm-film-thickness DB-624 capillary column (Agilent Technologies, Boulder, CO, USA). The GC oven temperature program had the following steps: 40°C for 10 min and 8°C min^−1^ to 250°C, followed by 250°C for 15 min. The column flow was split to direct approximately 1.5 ml min^−1^ of the column flow to the MS instrument for compound identification and the remainder to the FID for compound quantification. Additional details on the instrument setup and sample collection were reported previously (65).

### Volatile organic compound identification.

VOCs were identified using retention index data and by comparing their spectra to the National Institute of Standards and Technology (NIST) spectral library (version 2.2, 10 June 2014; NIST, Gaithersburg, MD, USA) using Agilent Chemstation software version F.01.03.2357. Linear programmed retention indices (66) were calculated based on retention times of *n*-alkanes that were observed in standard runs. Mass spectra were found by averaging approximately five scans at the peak maximum, subtracting an averaged background signal, and searching for matching spectra in the library. Based on compounds present in the National Physical Laboratory (NPL) standard, a VOC had to be present at mixing ratios of approximately 100 ppt to give a large enough signal within the 2-liter samples for structural peak identification. Because of the approximately 7-times-higher sensitivity of the FID than of the MS instrument, more VOCs are reported for the profile analyses (see below) that are based on the FID than what could be structurally identified by the MS analyses. Two identified compounds coeluted at the same retention time of 31.1 min, 3-methyl-2-pentanone and dimethyl disulfide. For the quantitative VOC profile analyses (see below), these were treated as a single compound, but for qualitative descriptions, they were treated as distinct compounds.

### Volatile organic compound profile analyses.

All actinobacterial VOC profile analyses were performed using the presence/absence criterion. Only compounds with retention times of between 18.7 and 40 min and FID peak areas of >5,000 mVs were included. This peak area threshold corresponds to VOC mixing ratios of approximately 15 ppt. A compound was considered distinct based on its retention time, rounded to the nearest 10th decimal place. Finally, we removed VOCs that were consistently emitted by sterile medium samples (i.e., “medium blanks” or VOCs released from the media by abiotic processes), which included acetaldehyde, acetone, 2-methylpropanal, 2-butanone, 3-methylbutanal, and 2-methylbutanal. We detected a number of compounds in the ISP2 medium blanks that were not detected the GA medium blanks (including acetonitrile), and while these compounds were included in the GA medium VOC profile analyses, we acknowledge that these could represent sterile medium emissions (see Table S3 in the supplemental material).

Differences in VOC emission profiles between strains were visualized using nonmetric multidimensional scaling (NMDS) of Jaccard dissimilarities. Strains that did not produce any detectable VOCs were removed to minimize distance in the matrix. We used permutational analysis of variance (ANOVA) implemented with the R package “vegan” (67) to test for differences in VOC profiles between strains and growth conditions, and statistical significance was evaluated following 999 permutations. We used the R package “ade4” (68) to perform Mantel tests to determine the relationship between pairwise 16S rRNA gene sequence distances and VOC profile Jaccard dissimilarities, with statistical significance evaluated following 999 permutations.

### Pseudomonad growth assay.

We investigated the ecological relevance and functional potential of these mVOCs by evaluating differences in growth rates of two test pseudomonad strains in the presence of actinobacterial VOCs. These pseudomonads were chosen as they are plant-colonizing rhizobacteria that are considered either plant growth promoting (Pseudomonas fluorescens SBW25 [41]) or phytopathogenic (Pseudomonas syringae pv. tomato DC3000 [42]). We subsampled actinobacterial strains for this assay and further restricted our analyses to those strains that grew readily on ISP2 medium, and these 24 actinobacterial strains (see Table S1 in the supplemental material) were plated onto ISP2 agar and incubated at 30°C for 12 to 14 days. Cells were harvested by washing plates with 0.1% Tween 20 (Sigma-Aldrich, St. Louis, MO, USA), centrifuging at 12,000 × *g* for 5 min, and suspending cell pellets in 1 ml 0.1× phosphate-buffered saline (PBS). Twenty microliters of the cell suspension was transferred into the wells of a Nunc MicroWell 96-well microplate (Thermo Fisher Scientific, Waltham, MA, USA) containing 300 µl of ISP2 agar (32 replicate wells [A1 to A8, C1 to C8, E1 to E8, and G1 to G8]). Culture plates were incubated at 30°C for 5 days to ensure VOC production.

Pseudomonad strains were grown overnight at 30°C with shaking (180 rpm) in King’s B medium (Table S2). Cultures grown overnight were diluted into fresh medium to an optical density (OD) (at 600 nm) of 0.05, and 250 µl of diluted pseudomonad cultures was transferred to the culture plate containing the actinobacterial cultures (8 replicate wells per strain [B1 to B8 for P. fluorescens SBW25 and D1 to D8 for P. syringae pv. tomato DC3000]). Thus, the pseudomonad cultures were exposed to actinobacterial volatiles that diffused across the shared headspace of the culture plate throughout the growth assay. Growth rates of pseudomonad test strains were measured using a Synergy HT microplate reader (BioTek, Winooski, VT, USA) at 24°C with slow, continuous shaking. The absorbance at 630 nm was measured every 20 min for a total of 980 min. The absorbance values of sterile King’s B medium (i.e., medium blanks) were subtracted from each measurement.

Growth rates were determined using the R package “Growthcurver” (69). We asked whether the mean growth rate of pseudomonad strains (averaged across the 8 replicates per growth curve) exposed to the volatiles from each actinobacterial strain differed from the mean growth rate of pseudomonad test strains in the presence of sterile ISP2 medium blanks (*t* test without *P* value adjustment). For each growth curve, we excluded replicates with a poor fit to the logistic curve and also excluded outliers with sigma values of ≤0.08 (see Growthcurver documentation), which resulted in the removal of strain FLCC682 from P. fluorescens SBW25 analyses. Mean pseudomonad control growth rates (i.e., ISP2 medium blanks) were averaged across independent growth curve experiments, and growth rates were equivalent across biological replicates (*P > *0.05 by a *t* test). We randomly chose two actinobacterial strains (FLCC45 and FLCC517) for independent validation of this method, and mean pseudomonad growth rates across biological replicates were equivalent (*P > *0.05 by a *t* test). Finally, we used the R package “UpSetR” (70) to identify and visualize collections of VOCs correlated with inhibition or stimulation of pseudomonad growth.

### Data availability.

All actinobacterial 16S rRNA gene sequences are provided in Data Set S1 in the supplemental material. NCBI accession numbers for whole genomes and marker gene sequences (when available) are listed in Table S1.

10.1128/mSystems.00295-18.9 DATA SET S1 All actinobacterial DNA sequences provided in a fasta file, which contains the multiple-sequence alignment of partial 16S rRNA gene sequences used to construct the phylogeny in Fig. 1. Sequences were aligned using MAFFT (60), and poorly aligned regions were removed with trimAL (61), resulting in an aligned nucleotide fragment of 682 bp. Sequences are labeled with strain names (see Table S1 in the supplemental material). The alignment also includes Bifidobacterium bifidum JCM 1255 (Bif_bif_JCM1255) and Bifidobacterium breve KSS01 (Bif_breve_KSS01) sequences. Download Data Set S1, TXT file, 0.03 MB. Copyright © 2019 Choudoir et al. 2019 Choudoir et al. This content is distributed under the terms of the Creative Commons Attribution 4.0 International license.
