# Supplementary material for: A Phylogenetic and Functional Perspective on Volatile Organic Compound Production by Actinobacteria
Source: mSystems. 2019 Mar 5;4(2):e00295-18. doi: 10.1128/mSystems.00295-18 (PMC6401417; doi:10.1128/mSystems.00295-18)
Supplement: TABLE S1 [file mSystems.00295-18-st001.docx]

| Strain | Family | Genus | Species | Culture Collection | Isolation Media | Source | NCBI Accession |
| --- | --- | --- | --- | --- | --- | --- | --- |
| B16927* | *Streptomycetaceae* | *Streptomyces* | *atratus* | ARS NRRL | unknown | unknown | NR_043490 |
| B2000* | *Streptomycetaceae* | *Streptomyces* | *anulats* | ARS NRRL | unknown | unknown | NR_043489 |
| B2682* | *Streptomycetaceae* | *Streptomyces* | *griesus* | ARS NRRL | unknown | unknown | SAMN05558834 |
| B2690* | *Streptomycetaceae* | *Streptomyces* | *bikiniensis* | ARS NRRL | unknown | unknown | SAMN02645389 |
| B2808* | *Streptomycetaceae* | *Streptomyces* | *aureus* | ARS NRRL | unknown | unknown | SAMN04002963 |
| CLCC1251 | *Nocardioidaceae* | *Kribella* |  | Fierer Lab | RM | sub-alpine forest soil |  |
| CLCC1393* | *Nocardiaceae* | *Rhodococcus* |  | Fierer Lab | RM | sub-alpine forest soil |  |
| CLCC1488 | *Pseudonocardiaceae* | *Amycolatopsis* |  | Fierer Lab | RM | sub-alpine forest soil |  |
| CLCC371A | *Nocardioidaceae* | *Nocardioides* |  | Fierer Lab | RM | sub-alpine forest soil |  |
| CLCC460 | *Pseudonocardiaceae* | *Amycolatopsis* |  | Fierer Lab | RM | sub-alpine forest soil |  |
| CLCC501 | *Mycobacteriaceae* | *Mycobacterium* |  | Fierer Lab | RM | sub-alpine forest soil |  |
| CLCC567* | *Microbacteriaceae* | *Clavibacter* | *michiganensis* | Fierer Lab | RM | sub-alpine forest soil |  |
| CLCC686 | *Streptomycetaceae* |  |  | Fierer Lab | RM | sub-alpine forest soil |  |
| CLCC811 | *Streptomycetaceae* | *Streptomyces* |  | Fierer Lab | RM | sub-alpine forest soil |  |
| CLCC866 | *Actinosynnemataceae* | *Kibdelosporangium* |  | Fierer Lab | RM | sub-alpine forest soil |  |
| FLCC167 | *Microbacteriaceae* | *Clavibacter* | *michiganensis* | Fierer Lab | CN Gellan | airborne dust |  |
| FLCC191* | *Micrococcaceae* | *Microbispora* |  | Fierer Lab | CN Gellan | airborne dust |  |
| FLCC204 | *Promicromonosporaceae* | *Promicromonospora* |  | Fierer Lab | CN Gellan | airborne dust |  |
| FLCC211 | *Micrococcaceae* | *Kocuria* |  | Fierer Lab | CN Gellan | airborne dust |  |
| FLCC251* | *Mycobacteriaceae* | *Mycobacterium* |  | Fierer Lab | CN Gellan | airborne dust |  |
| FLCC270* | *Mycobacteriaceae* | *Mycobacterium* |  | Fierer Lab | CN Gellan | airborne dust |  |
| FLCC291* | *Promicromonosporaceae* | *Promicromonospora* |  | Fierer Lab | CN Gellan | airborne dust |  |
| FLCC336 | *Microbacteriaceae* | *Rathayibacter* | *caricis* | Fierer Lab | CN Gellan | airborne dust |  |
| FLCC365 | *Micromonosporaceae* |  |  | Fierer Lab | CN Gellan | airborne dust |  |
| FLCC378 | *Nocardiaceae* | *Rhodococcus* |  | Fierer Lab | CN Gellan | airborne dust |  |
| FLCC382 | *Micrococcaceae* |  |  | Fierer Lab | CN Gellan | airborne dust |  |
| FLCC400 | *Nocardioidaceae* | *Aeromicrobium* |  | Fierer Lab | CN Gellan | airborne dust |  |
| FLCC424 | *Microbacteriaceae* | *Frigoribacterium* |  | Fierer Lab | CN Gellan | airborne dust |  |
| FLCC425 | *Williamsiaceae* | *Williamsia* |  | Fierer Lab | CN Gellan | airborne dust |  |
| FLCC45* | *Nocardiaceae* | *Rhodococcus* |  | Fierer Lab | CN Gellan | airborne dust |  |
| FLCC483* | *Brevibacteriaceae* | *Brevibacterium* |  | Fierer Lab | ISP2 | airborne dust |  |
| FLCC487 | *Dietziaceae* | *Dietzia* |  | Fierer Lab | ISP2 | airborne dust |  |
| FLCC517* | *Micrococcaceae* | *Kocuria* |  | Fierer Lab | ISP2 | airborne dust |  |
| FLCC536* | *Microbacteriaceae* | *Curtobacterium* |  | Fierer Lab | ISP2 | airborne dust |  |
| FLCC572* | *Dietziaceae* | *Dietzia* |  | Fierer Lab | ISP2 | airborne dust |  |
| FLCC580 | *Corynebacteriaceae* | *Corynebacterium* |  | Fierer Lab | ISP2 | airborne dust |  |
| FLCC594* | *Streptomycetaceae* | *Streptomyces* |  | Fierer Lab | ISP2 | airborne dust |  |
| FLCC627* | *Micrococcaceae* | *Citricoccus* |  | Fierer Lab | ISP2 | airborne dust |  |
| FLCC649 | *Micrococcaceae* | *Citricoccus* | *alkalitolerans* | Fierer Lab | ISP2 | airborne dust |  |
| FLCC662 | *Brevibacteriaceae* | *Brevibacterium* |  | Fierer Lab | ISP2 | airborne dust |  |
| FLCC663* | *Dietziaceae* | *Dietzia* |  | Fierer Lab | ISP2 | airborne dust |  |
| FLCC678 | *Brevibacteriaceae* | *Brevibacterium* |  | Fierer Lab | ISP2 | airborne dust |  |
| FLCC682* | *Microbacteriaceae* | *Agrococcus* | *jenensis* | Fierer Lab | ISP2 | airborne dust |  |
| FLCC712* | *Microbacteriaceae* |  |  | Fierer Lab | ISP2 | airborne dust |  |
| ms115* | *Streptomycetaceae* | *Streptomyces* | *griesus* | Buckley Lab | GA | grassland soil | SAMN07606150 |
| or3* | *Streptomycetaceae* | *Streptomyces* | *griesus* | Buckley Lab | GA | grassland soil | SAMN07606152 |
| t99* | *Streptomycetaceae* | *Streptomyces* | *griesus* | Buckley Lab | GA | grassland soil | SAMN07606163 |
| wa1063 | *Streptomycetaceae* | *Streptomyces* | *griesus* | Buckley Lab | GA | grassland soil | SAMN07606165 |
