# Supplementary material for: A Phylogenetic and Functional Perspective on Volatile Organic Compound Production by Actinobacteria
Source: mSystems. 2019 Mar 5;4(2):e00295-18. doi: 10.1128/mSystems.00295-18 (PMC6401417; doi:10.1128/mSystems.00295-18)
Supplement: TABLE S2 [file mSystems.00295-18-st002.docx]

| **CN Gellan**^a^ |  |  |
| --- | --- | --- |
| Casamino acids | 1 | g |
| Nutrient broth (Difco) | 1 | g |
| Gellan gum | 12 | g |
| H_2_O | 1000 | mL |
| *Autoclave* |  |  |
|  |  |  |
| **Glycerol Arginine (GA)**^b^ |  |  |
| Glycerol | 12.5 | g |
| Arginine | 1 | g |
| NaCl | 1 | g |
| K_2_HPO_4_ | 1 | g |
| MgSO_4_ 7Η_2_O | 0.5 | g |
| Fe_2_(SO_4_)_3_ 6H_2_O | 0.01 | g |
| CuSO_4_ 5H_2_O | 0.001 | g |
| ZnSO_4_ 7H_2_O | 0.001 | g |
| MnSO_4_ H_2_O | 0.001 | g |
| Agar | 15 | g |
| H_2_O | 1000 | mL |
| *Adjust pH to 8.7, autoclave* |  |  |
|  |  |  |
| **ISP2**^c^ |  |  |
| Yeast extract | 4 | g |
| Malt extract | 10 | g |
| Dextrose | 4 | g |
| Arginine* | 2.5 | g |
| Agar | 20 | g |
| H20 | 1000 | mL |
| *Autoclave* |  |  |
| **Arginine was added to this recipe to stimulate sporulation* | | |
|  |  |  |
| **King's B *Pseudomonas* Media**^d^ |  |  |
| Peptone | 20 | g |
| Glycerol | 10 | g |
| K_2_HPO_4_ | 1.5 | g |
| Agar | 15 | g |
| H_2_0 | 1000 | mL |
| *Adjust pH to 7.2, autoclave, adjust to 1 M MgSO_4_* | |  |
|  |  |  |
| **Robin's Media (RM)** |  |  |
| ***Salts*** |  |  |
| 2-(N-morpholino)ethanesulfonic acid | 1.95 | g |
| MgSO_4_ | 0.02 | g |
| CaCl_2_ | 0.03 | g |
| ***Amino Acids*** |  |  |
| Serine | 0.03 | g |
| Glycine | 0.01 | g |
| Homocysteine | 0.03 | g |
| Isoleucine | 0.03 | g |
| Valine | 0.03 | g |
| Leucine | 0.03 | g |
| Arginine | 0.04 | g |
| Histidine | 0.04 | g |
| Tryptophan | 0.05 | g |
| Phenylalanine | 0.04 | g |
| Tyrosine | 0.05 | g |
| ***Carbon Source*** |  |  |
| Malt extract | 1 | g |
| Agar | 7.5 | g |
| H_2_O | 1000 | mL |
| *Adjust pH to 5.0, autoclave* |  |  |
| ***After autoclaving, add:*** |  |  |
| Ammonium phosphate solution | 1 | mL |
| Selenite-tungstate solution | 1 | mL |
| Vitamin solution 1 | 1 | mL |
| Vitamin solution 2 | 3 | mL |
| Trace element solution SL-10 + lanthanum | 1 | mL |

| ^a^Gavrish E, Bollmann A, Epstein S, Lewis K. 2008. A trap for *in situ* cultivation of filamentous actinobacteria. J Microbiol Methods 72:257–262. |
| --- |
| ^b^El-Nakeeb MA, Lechevalier HA. 1963. Selective isolation of aerobic Actinomycetes. Appl Microbiol 11:75–79.  ^c^Shirling EB, Gottlieb D. Methods for characterixation of *Streptomyces* species. 1966. Int J Syst Bacteriol 16:313–340.  ^d^King EO, Ward MK, Raney DE. Two simple media for the demonstration of pyocyanin and fluorescin. 1954. J Lab Clin Med 44:301–307. |
