# Supplementary material for: A Phylogenetic and Functional Perspective on Volatile Organic Compound Production by Actinobacteria
Source: mSystems. 2019 Mar 5;4(2):e00295-18. doi: 10.1128/mSystems.00295-18 (PMC6401417; doi:10.1128/mSystems.00295-18)
Supplement: TABLE S4 [file mSystems.00295-18-st004.docx]

| **Retention Time (min)** | **Retention Index** | **Compound ID** | **Peak Mass Spectrum^a^** | **Best Match Mass Spectrum** | **% Library match** |
| --- | --- | --- | --- | --- | --- |
| 19.6 |  | Methanol | 31 (999), 32 (760), 30 (60), 44 (57), 41 (6) | 31 (999), 32 (743), 29 (445), 15 (123), 30 (64) | 89.2 |
| 20.4 | 406.9 | Methanethiol | 47 (999), 48 (888), 45 (351), 41 (21), 37 (20) | 47 (999), 48 (899), 45 (474), 46 (115), 15 (96) | 94.6 |
| 20.7 | 417.2 | Trimethylamine | 58 (999), 59 (483), 42 (284), 30 (144), 57 (61) | 58 (999), 59 (466), 42 (351), 30 (134), 43 (81) | 83.7 |
| 20.9 | 424.1 | Ethanol | 31 (999), 45 (665), 44 (591), 46 (296), 43 (113) | 31 (999), 45 (572), 46 (245), 27 (176), 29 (119) | 76.3 |
| 21.6 | 448.3 | Acetonitrile* | 41 (999), 40 (519), 32 (316), 39 (101), 38 (42) | 41 (999), 40 (455), 39 (132), 14 (87), 38 (59) | 79.6 |
| 22.4 | 475.9 | Isopropyl alcohol | 45 (999), 43 (177), 44 (86), 42 (20), 41 (20) | 45 (999), 43 (142), 27 (93), 41 (62), 29 (53) | 6.87 |
| 23.4 | 508.6 | 2-Methyl-2-propanol | 59 (999), 31 (206), 41 (159), 43 (61), 39 (45) | 59 (999), 31 (377), 41 (257), 43 (167), 39 (111) | 79.6 |
| 23.5 | 511.4 | Acetic acid methyl ester | 43 (999), 74 (283), 59 (107), 42 (102), 44 (25) | 43 (999), 74 (214), 15 (150), 42 (79), 59 (64) | 85.8 |
| 24.1 | 528.6 | Nitromethane | 61 (999), 46 (515), 30 (456), 32 (326), 44 (238) | 30 (999), 61 (570), 15 (480), 46 (370), 29 (110) | 91.4 |
| 24.2 | 531.4 | Carbon disulfide | 76 (999), 32 (269), 44 (101), 78 (64), 53 (50) | 76 (999), 44 (163), 32 (142), 78 (75), 38 (47) | 98.6 |
| 25.4 | 565.7 | Acetic acid ethenyl ester | 43 (999), 86 (94), 44 (89), 32 (67), 42 (34) | 43 (999), 15 (110), 27 (67), 86 (65), 42 (63) | 82.0 |
| 26.1 | 585.7 | 2-Butanol | 45 (999), 59 (218), 43 (200), 44 (133), 31 (125) | 45 (999), 59 (225), 31 (142), 41 (112), 43 (101) | 70.4 |
| 27.0 | 603.0 | 2-Methyl-1-propanol | 43 (999), 41 (681), 42 (638), 33 (569), 39 (231) | 43 (999), 33 (679), 41 (679), 42 (679), 31 (572) | 91.7 |
| 28.0 | 642.4 | 3-Methyl-2-butanone | 43 (999), 86 (163), 41 (130), 39 (64), 44 (29) | 43 (999), 86 (211), 41 (152), 27 (79), 71 (60) | 63.4 |
| 28.1 | 645.5 | 1-Butanol | 56 (999), 41 (678), 43 (581), 42 (327), 55 (198) | 31 (999), 56 (992), 41 (918), 43 (827), 42 (548) | 62.8 |
| 28.8 | 666.7 | 2-Pentanone | 43 (999), 86 (163), 41 (101), 42 (67), 39 (61) | 43 (999), 86 (197), 41 (138), 58 (98), 71 (97) | 81.8 |
| 28.9 | 669.7 | 2,3-Pentanedione | 43 (999), 57 (444), 100 (216), 42 (52), 44 (49) | 43 (999), 29 (608), 57 (328), 27 (258), 15 (143) | 85.3 |
| 29.1 | 675.8 | 3-Pentanone | 57 (999), 86 (241), 32 (133), 44 (103) | 57 (999), 29 (593), 86 (211), 27 (123), 28 (42) | 95.4 |
| 29.3 | 681.8 | 3-Hydroxy-2-butanone | 45 (999), 43 (587), 42 (64), 88 (63), 44 (34) | 45 (999), 43 (533), 27 (69), 88 (64), 29 (58) | 48.7 |
| 29.7 | 693.9 | Acetic acid propyl ester | 43 (999), 61 (285), 44 (94), 42 (84), 41 (35) | 43 (999), 61 (226), 73 (99), 42 (78), 41 (55) | 61.5 |
| 30.1 | 706.7 | Butanoic acid methyl ester | 43 (999), 74 (928), 71 (469), 59 (124), 41 (103) | 43 (999), 74 (871), 71 (654), 41 (381), 59 (311) | 64.2 |
| 30.3 | 713.3 | Pyrazine* | 80 (999), 53 (382), 52 (136), 40 (54), 51 (54) | 80 (999), 53 (452), 26 (422), 52 (103), 51 (70) | 85.1 |
| 30.4 | 716.7 | 3-Methyl-1-butanol | 55 (999), 42 (772), 70 (674), 43 (631), 41 (578) | 55 (999), 42 (870), 70 (696), 43 (689), 41 (598) | 64.9 |
| 30.6 | 723.3 | 2-Methyl-1-butanol | 57 (999), 56 (865), 41 (813), 70 (416), 55 (248) | 41 (999), 57 (779), 56 (728), 29 (640), 31 (388) | 62.8 |
| 31.1 | 740.0 | 3-Methyl-2-pentanone | 43 (999), 57 (311), 41 (249), 72 (199), 56 (133) | 43 (999), 29 (337), 57 (274), 41 (262), 72 (171) | 82.7 |
| 31.1 | 740.0 | Dimethyldisulfide | 94 (999), 79 (489), 45 (451), 43 (324), 46 (271) | 94 (999), 79 (570), 45 (478), 46 (250), 47 (193) | 91.0 |
| 31.7 | 760.0 | 2,3-Hexanedione | 43 (999), 71 (160), 41 (142), 44 (52), 114 (17) | 43 (999), 41 (214), 71 (199), 27 (92), 114 (67) | 62.5 |
| 31.9 | 766.7 | 3-Hexanone | 43 (999), 57 (945), 71 (649), 100 (316), 41 (219) | 43 (999), 57 (848), 71 (539), 29 (529), 100 (285) | 82.0 |
| 32.0 | 770.0 | Cyclopentanone | 55 (999), 28 (370), 41 (340), 84 (250), 42 (240) | 55 (999), 28 (500), 84 (420), 41 (377), 56 (289) | 88.4 |
