# Supplementary material for: A Phylogenetic and Functional Perspective on Volatile Organic Compound Production by Actinobacteria
Source: mSystems. 2019 Mar 5;4(2):e00295-18. doi: 10.1128/mSystems.00295-18 (PMC6401417; doi:10.1128/mSystems.00295-18)
Supplement: TABLE S3 [file mSystems.00295-18-st003.pdf]

Retention Index/Retention Time (min)

										400.0	403.4	406.9	410.3	413.8	417.2	420.7	424.1	427.6	434.5	437.9	448.3	451.7	455.2	472.4	475.9	479.3	489.7	493.1	502.9	505.7	508.6	511.4	514.3	517.1	520.0	528.6	531.4	540.0	542.9	545.7	548.6	551.4	554.3	557.1
Strain	Media	Total # VOCs	18.7	18.8	19.1	19.6	19.7	19.8	20	20.2	20.3	20.4	20.5	20.6	20.7	20.8	20.9	21.0	21.2	21.3	21.6*	21.7	21.8	22.3	22.4	22.5	22.8	22.9	23.2	23.3	23.4	23.5	23.6	23.7	23.8	24.1	24.2	24.5*	24.6	24.7	24.8	24.9	25.0	25.1
B16927	GA	17	0	1	0	0	0	0	1	0	0	0	0	0	0	0	0	0	0	0	0	0	0	0	1	0	0	0	0	0	1	0	0	0	0	1	0	0	0	0	0	0	0	0
B16927	ISP2	25	0	0	0	1	0	0	0	0	0	1	0	0	1	0	1	0	0	0	0	0	0	0	1	0	0	0	0	0	1	0	0	0	0	1	0	0	1	0	0	0	0	0
B2000	GA	13	0	1	0	1	0	0	0	0	0	0	0	0	0	0	0	0	0	0	1	0	0	0	1	0	0	0	1	0	1	0	0	0	0	1	0	0	0	0	0	0	0	0
B2000	ISP2	20	0	1	0	1	0	0	0	0	0	1	0	0	0	0	1	0	0	0	0	0	0	0	1	0	0	0	1	0	1	0	0	0	0	1	0	0	0	0	0	0	0	0
B2682	GA	15	0	0	0	0	1	0	0	0	0	1	0	0	0	0	0	1	0	0	1	0	0	0	0	0	0	0	0	0	1	0	0	0	0	1	0	0	0	0	0	0	0	0
B2682	ISP2	25	0	0	0	1	0	0	0	0	0	1	1	0	0	0	1	0	0	0	0	0	0	0	1	0	0	0	0	0	1	0	0	0	0	1	0	0	1	0	0	0	0	0
B2690	GA	17	0	0	0	1	0	0	0	0	0	1	0	0	0	0	0	1	0	0	0	0	0	0	1	0	0	0	0	0	1	0	0	0	0	1	0	0	0	0	0	0	0	0
B2690	ISP2	22	0	0	0	0	0	0	0	1	0	1	0	0	1	0	1	0	0	0	0	1	0	0	1	0	0	0	0	0	1	0	0	0	0	1	0	0	0	0	0	0	1	0
B2808	GA	22	1	0	0	1	0	0	0	0	0	1	0	0	0	0	0	0	0	0	1	0	0	0	1	0	0	0	0	0	1	0	0	0	0	1	0	0	0	0	0	0	1	0
B2808	ISP2	27	1	0	0	1	0	0	0	0	0	0	1	0	0	0	1	0	0	0	0	1	0	0	1	0	0	0	0	0	1	0	0	0	0	0	0	0	0	0	0	0	1	0
CLCC1251	GA	11	0	0	0	0	0	0	0	0	0	0	0	0	0	0	0	0	0	0	0	0	0	0	1	0	0	0	0	0	1	0	0	0	0	1	0	1	0	0	0	0	0	0
CLCC1251	ISP2	14	0	0	0	0	0	0	0	0	0	0	0	0	0	0	0	0	0	0	0	0	0	0	1	0	0	0	1	0	1	0	0	0	0	1	0	0	0	0	0	0	0	0
CLCC1393	GA	3	0	0	0	0	0	0	0	0	0	0	0	0	0	0	0	1	0	0	0	0	0	0	0	0	0	0	0	0	1	0	0	0	0	1	0	0	0	0	0	0	0	0
CLCC1393	ISP2	7	0	0	0	1	0	0	0	0	0	0	0	0	1	0	1	0	0	0	0	0	0	0	0	0	0	0	0	0	1	0	0	0	0	1	0	0	0	0	0	0	0	0
CLCC1488	GA	12	0	1	0	0	0	1	0	0	0	0	0	0	0	0	1	0	0	0	0	0	0	1	0	1	0	0	0	1	0	0	0	0	0	0	0	0	0	0	0	0	0	1
CLCC1488	ISP2	NA	NA	NA	NA	NA	NA	NA	NA	NA	NA	NA	NA	NA	NA	NA	NA	NA	NA	NA	NA	NA	NA	NA	NA	NA	NA	NA	NA	NA	NA	NA	NA	NA	NA	NA	NA	NA	NA	NA	NA	NA	NA	NA
CLCC371A	GA	15	0	0	0	0	0	0	0	0	0	1	0	0	0	0	0	0	0	0	1	0	0	0	0	0	0	0	0	0	1	0	0	0	0	0	0	1	0	0	0	0	0	0
CLCC371A	ISP2	15	0	1	0	1	0	0	0	0	0	0	0	0	0	0	1	0	0	0	0	1	0	0	1	0	0	0	0	0	0	1	0	0	0	1	0	0	0	0	0	0	0	0
CLCC460	GA	18	0	0	0	0	0	0	0	0	0	0	0	0	0	0	0	0	0	0	0	0	0	0	1	0	0	0	0	0	0	0	0	0	0	1	0	1	0	0	0	0	0	0
CLCC460	ISP2	23	0	0	0	0	0	0	0	0	0	0	0	1	0	0	1	0	0	0	0	1	0	0	1	0	0	0	0	0	1	0	0	0	0	1	1	0	0	0	0	1	0	0
CLCC501	GA	4	0	0	0	0	1	0	0	0	0	0	0	0	0	0	0	0	1	0	0	0	0	0	0	1	0	0	0	0	0	0	0	0	0	0	0	0	0	0	0	0	0	0
CLCC501	ISP2	10	0	1	0	0	1	0	0	0	0	0	0	0	0	0	1	0	0	0	0	0	0	0	0	1	0	0	1	0	1	0	0	0	0	1	0	0	0	0	0	0	0	0
CLCC567	GA	NA	NA	NA	NA	NA	NA	NA	NA	NA	NA	NA	NA	NA	NA	NA	NA	NA	NA	NA	NA	NA	NA	NA	NA	NA	NA	NA	NA	NA	NA	NA	NA	NA	NA	NA	NA	NA	NA	NA	NA	NA	NA	NA
CLCC567	ISP2	21	0	1	0	1	0	0	0	0	0	0	0	0	0	0	0	0	0	0	0	0	1	0	1	0	0	0	0	1	1	0	0	0	0	1	0	0	0	0	0	0	0	0
CLCC686	GA	22	1	0	0	0	1	0	0	0	0	1	0	0	0	0	0	0	0	0	1	0	0	0	1	0	0	0	1	0	1	0	0	0	0	1	0	1	0	0	0	0	0	0
CLCC686	ISP2	21	1	1	0	1	0	0	0	1	0	1	0	0	0	0	1	0	0	0	0	0	0	0	1	0	0	0	1	0	1	0	0	0	0	1	0	0	0	0	0	0	0	0
CLCC811	GA	18	0	0	0	0	1	0	0	0	0	0	0	0	0	0	0	0	0	0	0	0	0	0	1	0	0	0	1	0	0	0	0	0	0	1	0	0	0	0	0	0	0	0
CLCC811	ISP2	17	1	0	0	0	0	0	0	0	0	0	0	0	0	0	0	0	0	0	0	0	0	0	1	0	0	0	0	0	0	0	0	0	0	1	0	0	0	0	0	0	0	0
CLCC866	GA	17	1	1	0	0	0	0	1	1	0	0	0	0	0	0	0	0	0	0	1	0	0	0	1	0	0	0	0	0	1	0	0	0	0	0	0	1	0	0	0	0	0	0
CLCC866	ISP2	15	0	0	0	0	0	0	1	0	0	0	0	0	0	0	0	0	0	0	0	0	0	0	1	0	0	0	0	0	1	0	0	0	0	0	0	0	0	0	0	1	0	0
FLCC167	GA	NA	NA	NA	NA	NA	NA	NA	NA	NA	NA	NA	NA	NA	NA	NA	NA	NA	NA	NA	NA	NA	NA	NA	NA	NA	NA	NA	NA	NA	NA	NA	NA	NA	NA	NA	NA	NA	NA	NA	NA	NA	NA	NA
FLCC167	ISP2	32	0	1	0	0	0	1	0	0	1	0	0	1	0	0	1	0	0	1	0	0	0	1	0	1	0	0	0	1	0	0	0	0	1	0	0	0	0	1	0	0	0	1
FLCC191	GA	NA	NA	NA	NA	NA	NA	NA	NA	NA	NA	NA	NA	NA	NA	NA	NA	NA	NA	NA	NA	NA	NA	NA	NA	NA	NA	NA	NA	NA	NA	NA	NA	NA	NA	NA	NA	NA	NA	NA	NA	NA	NA	NA
FLCC191	ISP2	19	0	0	0	1	0	0	0	0	0	0	0	0	0	0	0	1	0	0	0	0	0	0	1	0	0	0	0	0	1	0	0	0	0	1	0	0	0	0	0	0	1	0
FLCC204	GA	19	0	0	0	1	0	0	0	0	0	1	0	0	0	0	0	0	0	0	0	0	0	0	1	0	0	0	1	0	1	1	0	0	0	0	0	0	0	0	0	0	0	0
FLCC204	ISP2	32	0	0	0	1	0	0	0	0	0	1	1	0	0	0	1	0	0	0	0	1	0	0	1	0	0	0	0	0	1	0	1	0	0	1	0	0	0	0	0	1	0	0
FLCC211	GA	1	0	0	0	0	0	0	0	0	0	0	0	0	0	0	0	0	0	0	0	0	0	0	0	0	0	0	0	0	0	0	0	0	0	0	0	0	0	0	0	0	0	0
FLCC211	ISP2	0	0	0	0	0	0	0	0	0	0	0	0	0	0	0	0	0	0	0	0	0	0	0	0	0	0	0	0	0	0	0	0	0	0	0	0	0	0	0	0	0	0	0
FLCC251	GA	7	0	1	0	1	0	0	0	0	0	0	0	0	0	0	0	0	1	0	0	0	0	0	0	0	0	0	0	0	1	0	0	0	0	0	0	0	0	0	0	0	0	0
FLCC251	ISP2	11	0	1	0	1	0	0	0	0	0	0	0	0	0	0	0	0	0	0	0	0	0	0	1	0	0	0	0	0	1	1	0	0	0	0	0	0	0	0	0	0	0	0
FLCC270	GA	3	0	0	0	0	0	0	0	0	0	0	0	0	0	0	1	0	0	0	0	0	0	0	0	0	0	0	0	0	1	0	0	0	0	0	0	0	0	0	0	0	0	0
FLCC270	ISP2	2	0	0	0	0	0	0	0	0	0	0	0	0	0	0	1	0	0	0	0	0	0	0	0	0	0	0	0	0	1	0	0	0	0	0	0	0	0	0	0	0	0	0
FLCC291	GA	11	0	0	0	1	0	0	0	0	0	0	0	0	0	0	0	0	0	0	1	0	0	0	1	0	0	0	0	0	0	1	0	0	0	0	0	0	0	0	0	0	0	0
FLCC291	ISP2	14	0	1	0	0	0	0	0	0	0	0	1	0	0	0	0	0	0	0	0	0	0	0	1	0	0	0	0	0	0	1	0	0	0	1	0	0	0	0	0	0	0	0
FLCC336	GA	13	0	0	0	1	0	0	0	0	0	0	0	0	0	0	0	0	0	0	1	0	0	0	1	0	0	0	0	0	0	1	0	0	0	0	0	1	0	0	0	0	0	0
FLCC336	ISP2	15	0	0	0	1	0	0	0	0	0	1	0	0	0	0	0	0	0	0	0	0	0	0	1	0	0	0	0	0	0	1	0	0	0	0	0	0	0	0	0	0	0	0
FLCC365	GA	15	0	0	0	1	0	0	0	0	0	0	0	0	0	0	0	0	0	0	1	0	0	0	1	0	0	0	0	0	0	1	0	0	0	0	0	1	0	0	0	0	0	0
FLCC365	ISP2	23	0	0	0	1	0	0	0	0	0	1	0	0	0	0	1	0	0	0	0	1	0	0	1	0	0	0	0	0	0	1	0	0	0	0	0	0	0	0	0	0	0	0
FLCC378	GA	NA	NA	NA	NA	NA	NA	NA	NA	NA	NA	NA	NA	NA	NA	NA	NA	NA	NA	NA	NA	NA	NA	NA	NA	NA	NA	NA	NA	NA	NA	NA	NA	NA	NA	NA	NA	NA	NA	NA	NA	NA	NA	NA
FLCC378	ISP2	0	0	0	0	0	0	0	0	0	0	0	0	0	0	0	0	0	0	0	0	0	0	0	0	0	0	0	0	0	0	0	0	0	0	0	0	0	0	0	0	0	0	0
FLCC382	GA	1	0	0	0	0	0	0	0	0	0	0	0	0	0	0	0	0	0	0	0	0	0	0	0	0	0	0	0	0	0	0	0	0	0	0	0	0	0	0	0	0	0	0
FLCC382	ISP2	0	0	0	0	0	0	0	0	0	0	0	0	0	0	0	0	0	0	0	0	0	0	0	0	0	0	0	0	0	0	0	0	0	0	0	0	0	0	0	0	0	0	0
FLCC400	GA	28	0	1	1	0	0	1	0	0	0	1	0	0	0	0	1	0	0	1	0	0	0	1	0	1	0	1	0	1	0	0	0	0	0	0	0	0	0	1	0	0	0	1
FLCC400	ISP2	NA	NA	NA	NA	NA	NA	NA	NA	NA	NA	NA	NA	NA	NA	NA	NA	NA	NA	NA	NA	NA	NA	NA	NA	NA	NA	NA	NA	NA	NA	NA	NA	NA	NA	NA	NA	NA	NA	NA	NA	NA	NA	NA
FLCC424	GA	NA	NA	NA	NA	NA	NA	NA	NA	NA	NA	NA	NA	NA	NA	NA	NA	NA	NA	NA	NA	NA	NA	NA	NA	NA	NA	NA	NA	NA	NA	NA	NA	NA	NA	NA	NA	NA	NA	NA	NA	NA	NA	NA
FLCC424	ISP2	16	0	1	0	0	0	0	1	0	0	0	0	0	0	0	0	0	0	0	0	0	0	0	1	0	0	0	0	0	1	0	0	0	0	1	0	0	0	0	0	0	0	0
FLCC425	GA	1	0	0	0	0	0	0	0	0	0	0	0	0	0	0	0	0	0	0	0	0	0	0	0	0	0	0	0	0	1	0	0	0	0	0	0	0	0	0	0	0	0	0
FLCC425	ISP2	4	0	1	0	0	0	0	0	0	0	0	0	0	0	0	0	1	0	0	0	0	0	0	0	0	0	0	0	0	1	0	0	0	0	0	0	0	0	0	0	0	0	0
FLCC45	GA	NA	NA	NA	NA	NA	NA	NA	NA	NA	NA	NA	NA	NA	NA	NA	NA	NA	NA	NA	NA	NA	NA	NA	NA	NA	NA	NA	NA	NA	NA	NA	NA	NA	NA	NA	NA	NA	NA	NA	NA	NA	NA	NA
FLCC45	ISP2	6	0	0	0	0	1	0	0	0	0	0	0	0	0	0	0	0	0	0	0	0	0	0	1	0	0	0	0	0	1	0	0	0	0	0	0	0	0	0	0	0	0	0
FLCC483	GA	NA	NA	NA	NA	NA	NA	NA	NA	NA	NA	NA	NA	NA	NA	NA	NA	NA	NA	NA	NA	NA	NA	NA	NA	NA	NA	NA	NA	NA	NA	NA	NA	NA	NA	NA	NA	NA	NA	NA	NA	NA	NA	NA
FLCC483	ISP2	4	0	0	0	0	1	0	0	0	0	0	0	0	0	0	0	0	1	0	0	0	0	0	0	0	0	0	0	0	1	0	0	0	0	0	0	0	0	0	0	0	0	0
FLCC487	GA	NA	NA	NA	NA	NA	NA	NA	NA	NA	NA	NA	NA	NA	NA	NA	NA	NA	NA	NA	NA	NA	NA	NA	NA	NA	NA	NA	NA	NA	NA	NA	NA	NA	NA	NA	NA	NA	NA	NA	NA	NA	NA	NA
FLCC487	ISP2	16	0	1	0	0	0	1	0	0	0	1	0	0	0	0	1	0	0	1	0	0	0	0	0	1	0	0	0	0	0	0	0	0	0	0	0	0	0	0	0	0	0	1
FLCC517	GA	20	0	1	0	0	0	1	0	0	1	0	0	0	0	0	1	0	0	1	0	0	0	0	0	1	0	0	0	1	0	0	0	0	0	0	0	0	0	0	0	0	0	1
FLCC517	ISP2	25	0	1	0	0	0	1	0	0	1	0	0	0	0	0	1	0	0	0	0	0	0	0	0	1	0	0	1	0	0	0	0	0	0	0	0	0	0	1	1	0	0	1
FLCC536	GA	NA	NA	NA	NA	NA	NA	NA	NA	NA	NA	NA	NA	NA	NA	NA	NA	NA	NA	NA	NA	NA	NA	NA	NA	NA	NA	NA	NA	NA	NA	NA	NA	NA	NA	NA	NA	NA	NA	NA	NA	NA	NA	NA
FLCC536	ISP2	22	0	0	0	0	0	0	1	0	0	0	1	0	0	0	0	0	0	0	0	0	0	0	1	0	1	0	0	0	1	0	0	0	0	1	0	0	0	0	0	1	0	0
FLCC572	GA	NA	NA	NA	NA	NA	NA	NA	NA	NA	NA	NA	NA	NA	NA	NA	NA	NA	NA	NA	NA	NA	NA	NA	NA	NA	NA	NA	NA	NA	NA	NA	NA	NA	NA	NA	NA	NA	NA	NA	NA	NA	NA	NA
FLCC572	ISP2	6	0	1	0	0	1	0	0	0	0	0	0	0	0	0	0	0	1	0	0	0	0	0	0	1	0	0	0	0	1	0	0	0	0	0	0	0	0	0	0	0	0	0
FLCC580	GA	NA	NA	NA	NA	NA	NA	NA	NA	NA	NA	NA	NA	NA	NA	NA	NA	NA	NA	NA	NA	NA	NA	NA	NA	NA	NA	NA	NA	NA	NA	NA	NA	NA	NA	NA	NA	NA	NA	NA	NA	NA	NA	NA
FLCC580	ISP2	19	0	0	0	0	1	0	0	0	0	0	0	0	0	0	0	0	0	0	0	1	0	0	1	0	0	0	0	0	1	0	0	0	0	1	0	0	0	0	0	0	0	0
FLCC594	GA	17	0	0	0	0	0	0	0	0	0	0	0	0	0	0	0	0	0	0	0	0	0	0	1	0	0	0	1	0	0	0	0	0	0	1	0	1	0	0	0	0	0	0
FLCC594	ISP2	22	0	0	0	0	0	0	0	0	0	1	0	0	0	0	0	0	0	0	0	0	0	0	1	0	0	0	1	0	0	0	0	1	0	1	0	0	0	0	0	0	0	0
FLCC627	GA	NA	NA	NA	NA	NA	NA	NA	NA	NA	NA	NA	NA	NA	NA	NA	NA	NA	NA	NA	NA	NA	NA	NA	NA	NA	NA	NA	NA	NA	NA	NA	NA	NA	NA	NA	NA	NA	NA	NA	NA	NA	NA	NA
FLCC627	ISP2	8	0	0	0	1	0	0	0	0	0	0	0	0	0	0	0	0	0	0	0	0	0	0	1	0	0	0	0	0	0	1	0	0	0	0	0	0	0	0	0	0	0	0
FLCC649	GA	NA	NA	NA	NA	NA	NA	NA	NA	NA	NA	NA	NA	NA	NA	NA	NA	NA	NA	NA	NA	NA	NA	NA	NA	NA	NA	NA	NA	NA	NA	NA	NA	NA	NA	NA	NA	NA	NA	NA	NA	NA	NA	NA
FLCC649	ISP2	10	0	0	0	1	0	0	0	0	0	0	0	0	0	0	0	0	1	0	0	0	0	0	0	0	0	0	0	0	1	0	0	0	0	0	0	0	0	0	0	0	0	0
FLCC662	GA	NA	NA	NA	NA	NA	NA	NA	NA	NA	NA	NA	NA	NA	NA	NA	NA	NA	NA	NA	NA	NA	NA	NA	NA	NA	NA	NA	NA	NA	NA	NA	NA	NA	NA	NA	NA	NA	NA	NA	NA	NA	NA	NA
FLCC662	ISP2	0	0	0	0	0	0	0	0	0	0	0	0	0	0	0	0	0	0	0	0	0	0	0	0	0	0	0	0	0	0	0	0	0	0	0	0	0	0	0	0	0	0	0
FLCC663	GA	NA	NA	NA	NA	NA	NA	NA	NA	NA	NA	NA	NA	NA	NA	NA	NA	NA	NA	NA	NA	NA	NA	NA	NA	NA	NA	NA	NA	NA	NA	NA	NA	NA	NA	NA	NA	NA	NA	NA	NA	NA	NA	NA
FLCC663	ISP2	4	0	1	0	0	1	0	0	0	0	0	0	0	0	0	0	0	1	0	0	0	0	0	0	0	0	0	0	0	1	0	0	0	0	0	0	0	0	0	0	0	0	0
FLCC678	GA	NA	NA	NA	NA	NA	NA	NA	NA	NA	NA	NA	NA	NA	NA	NA	NA	NA	NA	NA	NA	NA	NA	NA	NA	NA	NA	NA	NA	NA	NA	NA	NA	NA	NA	NA	NA	NA	NA	NA	NA	NA	NA	NA
FLCC678	ISP2	16	0	1	0	0	0	1	0	0	1	0	0	0	0	0	1	0	0	1	0	0	0	0	0	1	0	0	0	1	0	0	0	0	0	0	0	0	0	0	0	1	0	1
FLCC682	GA	NA	NA	NA	NA	NA	NA	NA	NA	NA	NA	NA	NA	NA	NA	NA	NA	NA	NA	NA	NA	NA	NA	NA	NA	NA	NA	NA	NA	NA	NA	NA	NA	NA	NA	NA	NA	NA	NA	NA	NA	NA	NA	NA
FLCC682	ISP2	19	0	1	0	1	0	0	0	0	0	1	1	0	0	0	1	0	0	0	0	1	0	0	0	0	0	0	0	0	1	0	0	0	0	1	0	0	0	0	0	0	0	0
FLCC712	GA	NA	NA	NA	NA	NA	NA	NA	NA	NA	NA	NA	NA	NA	NA	NA	NA	NA	NA	NA	NA	NA	NA	NA	NA	NA	NA	NA	NA	NA	NA	NA	NA	NA	NA	NA	NA	NA	NA	NA	NA	NA	NA	NA
FLCC712	ISP2	17	0	1	0	0	0	0	0	0	0	0	0	0	0	0	0	0	0	0	0	0	0	0	1	0	0	0	0	0	1	0	0	0	0	0	0	0	0	0	0	0	0	0
ms115	GA	22	0	0	0	0	1	0	0	0	0	1	0	0	0	0	1	0	0	0	1	0	0	0	1	0	0	0	0	0	1	0	0	0	0	1	0	0	0	0	0	0	0	0
ms115	ISP2	22	0	1	0	1	0	0	0	1	0	1	0	0	0	0	1	0	0	0	0	0	0	0	1	0	0	0	0	0	1	0	0	0	0	1	0	0	0	0	0	0	0	0
or3	GA	31	0	0	0	0	0	0	1	0	0	1	0	0	0	0	1	0	0	0	1	0	0	0	1	0	0	0	0	0	1	0	0	0	0	1	0	0	1	0	0	0	0	0
or3	ISP2	25	0	0	0	1	0	0	0	0	0	1	0	0	0	0	1	0	0	0	0	0	0	0	1	0	0	0	0	0	1	0	0	0	0	1	0	0	1	0	0	0	0	0
t99	GA	21	0	0	0	0	1	0	0	0	0	1	0	0	0	0	1	0	0	0	1	0	0	0	1	0	0	0	0	0	1	0	0	0	0	1	0	0	1	0	0	0	0	0
t99	ISP2	32	0	0	0	0	1	0	0	0	0	1	0	0	1	1	0	0	0	0	0	1	0	0	1	0	0	0	1	0	1	0	0	0	1	1	0	0	1	0	0	0	0	0
wa1063	GA	20	0	0	0	0	1	0	0	0	0	1	0	0	0	0	1	0	0	0	1	0	0	0	0	1	0	0	0	1	1	0	0	0	0	1	0	0	1	0	0	0	0	0
wa1063	ISP2	24	0	0	0	0	0	0	1	0	0	1	0	0	0	0	1	0	0	0	0	1	0	0	1	0	0	0	0	0	1	0	0	0	0	1	0	0	1	0	0	0	0	0
**Sample Presence**			6	25	1	26	14	7	7	4	4	25	6	2	4	1	28	5	6	5	13	10	1	3	46	11	1	1	12	7	50	10	1	1	2	39	1	8	8	3	1	5	4	7

		565.7	568.6	571.4	580.0	582.9	585.7	588.6	594.3	597.1	600.0	603.0	612.1	615.2	624.2	627.3	630.3	633.3	636.4	642.4	645.5	648.5	654.5	657.6	660.6	663.6	666.7	669.7	672.7	675.8	678.8	681.8	693.9	697.0	700.0	703.3	706.7	710.0	713.3	716.7	723.3	726.7	733.3	736.7	740.0
Strain	Media	25.4	25.5	25.6	25.9	26.0	26.1	26.2	26.4	26.5	26.6	26.7	27.0	27.1	27.4	27.5	27.6	27.7	27.8*	28.0	28.1	28.2*	28.4	28.5	28.6	28.7	28.8	28.9	29.0	29.1	29.2	29.3	29.7	29.8	29.9	30.0	30.1	30.2	30.3*	30.4	30.6	30.7	30.9	31	31.1
B16927	GA	0	0	0	0	0	0	0	0	1	0	1	1	0	0	0	0	0	0	1	0	0	0	0	0	0	1	0	0	0	0	0	0	0	0	0	0	0	0	1	1	0	0	0	1
B16927	ISP2	0	1	0	0	1	0	0	0	1	0	1	1	0	0	0	0	0	0	1	0	0	0	0	0	0	1	1	0	0	0	0	0	0	0	0	0	0	0	1	1	0	0	0	1
B2000	GA	0	0	0	0	0	0	0	0	0	0	0	0	0	0	0	0	0	0	1	0	0	0	0	0	0	1	0	0	0	0	0	0	0	0	0	0	0	0	1	1	0	0	0	1
B2000	ISP2	0	0	0	0	1	0	0	0	0	1	0	1	0	0	0	0	0	0	1	1	0	0	0	0	0	1	0	0	1	0	0	0	0	0	0	0	0	0	1	1	0	0	1	0
B2682	GA	0	0	0	0	0	0	0	0	1	0	0	1	0	0	0	0	0	0	1	0	0	0	0	0	0	1	0	0	0	1	0	0	0	0	0	0	0	0	1	1	0	0	0	1
B2682	ISP2	1	0	0	0	1	0	0	0	1	0	0	1	0	0	1	0	0	0	1	0	0	0	1	0	0	1	0	0	1	0	0	0	0	0	0	0	0	0	1	1	0	0	0	1
B2690	GA	0	0	0	0	0	0	0	0	1	0	0	1	0	0	0	0	1	0	1	0	0	0	0	0	0	1	0	0	0	0	0	0	0	0	0	0	0	1	1	1	0	0	0	1
B2690	ISP2	0	1	0	0	0	1	0	0	1	0	0	1	0	0	0	0	0	0	1	1	0	0	0	0	0	1	0	0	0	0	0	0	1	0	0	0	0	0	1	1	0	0	0	1
B2808	GA	0	0	0	0	1	0	0	0	1	0	0	1	0	0	0	0	0	0	1	0	0	0	1	0	0	1	0	0	1	0	0	0	0	0	0	0	0	0	1	1	0	0	0	1
B2808	ISP2	1	0	0	0	0	1	0	0	1	0	0	1	0	0	0	0	0	0	1	0	0	0	1	0	0	1	1	0	1	0	0	0	0	0	0	0	0	0	1	1	0	0	0	1
CLCC1251	GA	0	0	0	0	0	0	0	0	0	0	0	1	0	0	0	0	0	1	1	0	1	0	0	0	0	1	0	0	0	0	0	0	0	0	0	0	0	0	1	1	0	0	0	0
CLCC1251	ISP2	0	0	0	0	0	1	0	0	1	0	1	1	0	0	0	0	0	0	0	1	0	0	0	0	0	1	0	0	0	0	0	0	0	0	0	0	0	0	1	1	0	0	1	0
CLCC1393	GA	0	0	0	0	0	0	0	0	0	0	0	0	0	0	0	0	0	0	0	0	0	0	0	0	0	0	0	0	0	0	0	0	0	0	0	0	0	0	0	0	0	0	0	0
CLCC1393	ISP2	1	0	0	0	0	0	0	0	0	0	0	0	0	0	0	0	0	0	0	0	0	0	0	0	0	1	0	0	0	0	0	0	0	0	0	0	0	0	0	0	0	0	0	0
CLCC1488	GA	0	0	0	0	0	0	0	0	0	0	1	0	0	0	0	0	0	0	0	0	0	0	0	1	0	0	0	0	0	0	0	0	0	0	0	0	0	0	0	0	0	0	0	0
CLCC1488	ISP2	NA	NA	NA	NA	NA	NA	NA	NA	NA	NA	NA	NA	NA	NA	NA	NA	NA	NA	NA	NA	NA	NA	NA	NA	NA	NA	NA	NA	NA	NA	NA	NA	NA	NA	NA	NA	NA	NA	NA	NA	NA	NA	NA	NA
CLCC371A	GA	0	0	0	0	0	0	0	0	1	0	0	1	0	0	0	0	0	0	1	1	0	0	0	0	0	1	1	0	0	0	0	0	0	0	0	0	0	0	1	1	0	0	0	1
CLCC371A	ISP2	0	0	0	0	0	0	0	0	1	0	0	1	0	0	0	0	0	0	1	1	0	0	0	0	0	0	0	0	0	0	0	0	0	0	0	0	0	0	1	1	0	0	0	1
CLCC460	GA	0	0	0	0	1	0	0	0	1	0	1	1	0	0	0	0	0	0	1	1	0	0	0	0	0	1	0	0	1	0	1	1	0	0	0	0	0	0	1	1	0	0	0	1
CLCC460	ISP2	1	0	0	0	1	0	0	0	0	0	0	1	0	0	0	0	0	0	1	1	0	1	0	0	0	1	0	0	1	0	1	0	0	0	0	0	0	0	1	1	0	0	0	1
CLCC501	GA	0	0	0	0	0	0	0	0	0	0	0	0	0	0	0	0	0	0	0	0	0	0	0	0	0	0	0	0	0	0	0	0	0	0	0	0	0	0	0	0	0	0	0	0
CLCC501	ISP2	0	0	0	0	0	1	0	0	1	0	0	0	0	0	0	0	0	0	0	0	0	0	0	0	0	0	0	0	0	0	0	0	0	0	0	0	0	0	0	0	0	0	0	0
CLCC567	GA	NA	NA	NA	NA	NA	NA	NA	NA	NA	NA	NA	NA	NA	NA	NA	NA	NA	NA	NA	NA	NA	NA	NA	NA	NA	NA	NA	NA	NA	NA	NA	NA	NA	NA	NA	NA	NA	NA	NA	NA	NA	NA	NA	NA
CLCC567	ISP2	0	0	0	0	0	0	0	0	1	0	0	1	0	0	0	0	0	0	0	1	0	0	0	0	0	1	1	0	0	0	1	0	0	0	0	0	0	0	1	1	0	0	0	1
CLCC686	GA	0	0	0	0	0	0	0	0	1	0	0	1	0	0	0	0	0	0	1	1	0	0	0	0	0	1	0	0	0	1	0	0	0	0	0	1	0	0	1	1	0	0	0	1
CLCC686	ISP2	0	0	0	0	0	1	0	0	1	0	0	1	0	0	0	0	0	0	1	1	0	0	0	0	0	1	0	0	0	1	0	0	0	0	0	0	0	0	1	1	0	0	0	1
CLCC811	GA	0	0	0	0	0	0	0	0	1	0	0	1	0	0	0	0	0	0	1	0	0	0	1	0	0	1	1	0	1	0	0	0	0	1	0	0	0	0	1	1	0	0	1	0
CLCC811	ISP2	0	0	0	0	1	0	0	1	0	0	0	1	0	0	0	0	0	0	1	1	0	0	0	0	0	0	1	0	1	0	0	0	0	0	1	0	0	0	1	1	0	0	1	0
CLCC866	GA	0	0	0	0	0	0	0	0	1	0	0	1	0	1	0	0	0	1	0	1	0	0	0	0	0	1	0	0	0	0	0	0	0	0	0	0	0	0	1	1	0	0	0	1
CLCC866	ISP2	0	0	0	0	0	1	0	1	0	0	0	1	0	0	0	0	0	0	1	1	0	0	0	0	0	1	0	0	0	1	0	0	0	0	0	0	0	0	1	1	0	0	1	0
FLCC167	GA	NA	NA	NA	NA	NA	NA	NA	NA	NA	NA	NA	NA	NA	NA	NA	NA	NA	NA	NA	NA	NA	NA	NA	NA	NA	NA	NA	NA	NA	NA	NA	NA	NA	NA	NA	NA	NA	NA	NA	NA	NA	NA	NA	NA
FLCC167	ISP2	0	1	0	0	1	0	0	0	1	0	0	0	1	0	1	0	1	0	0	0	0	1	0	1	1	0	0	0	1	0	0	0	0	0	0	1	0	0	1	0	0	1	0	0
FLCC191	GA	NA	NA	NA	NA	NA	NA	NA	NA	NA	NA	NA	NA	NA	NA	NA	NA	NA	NA	NA	NA	NA	NA	NA	NA	NA	NA	NA	NA	NA	NA	NA	NA	NA	NA	NA	NA	NA	NA	NA	NA	NA	NA	NA	NA
FLCC191	ISP2	0	0	0	0	0	0	0	0	1	0	0	1	0	0	0	0	0	0	1	0	0	0	0	0	0	1	0	0	0	0	1	0	0	0	0	0	0	0	1	1	0	0	0	1
FLCC204	GA	0	0	0	0	0	1	0	0	1	0	0	1	0	0	0	0	0	0	1	1	0	0	0	0	0	0	1	0	0	1	0	0	0	0	0	0	0	0	1	1	0	0	0	1
FLCC204	ISP2	0	0	0	1	1	0	0	0	1	0	0	1	0	0	0	0	0	0	1	1	0	0	0	0	0	1	1	0	0	0	0	0	0	0	0	0	0	0	1	1	0	0	0	1
FLCC211	GA	0	0	0	0	0	0	0	0	0	0	0	0	0	0	0	0	0	0	0	0	0	0	0	0	0	0	0	0	0	0	0	0	0	0	0	0	0	1	0	0	0	0	0	0
FLCC211	ISP2	0	0	0	0	0	0	0	0	0	0	0	0	0	0	0	0	0	0	0	0	0	0	0	0	0	0	0	0	0	0	0	0	0	0	0	0	0	0	0	0	0	0	0	0
FLCC251	GA	0	0	0	0	0	0	0	0	1	0	0	0	0	0	0	0	0	0	0	0	0	0	0	0	0	0	0	0	0	1	0	0	0	0	0	0	0	0	0	0	0	0	0	1
FLCC251	ISP2	0	0	0	0	0	0	0	0	0	0	0	1	0	0	0	0	0	0	1	1	0	0	0	0	0	0	0	0	0	0	0	0	0	0	0	0	0	0	1	1	0	0	0	1
FLCC270	GA	0	0	0	0	0	0	0	0	0	0	0	0	0	0	0	0	0	0	0	0	0	0	0	0	0	0	0	0	0	0	0	0	0	0	0	0	0	1	0	0	0	0	0	0
FLCC270	ISP2	0	0	0	0	0	0	0	0	0	0	0	0	0	0	0	0	0	0	0	0	0	0	0	0	0	0	0	0	0	0	0	0	0	0	0	0	0	0	0	0	0	0	0	0
FLCC291	GA	0	0	0	0	0	0	0	0	0	0	0	1	0	0	0	0	0	0	0	1	0	0	0	0	0	0	1	0	0	0	0	0	0	0	0	0	0	0	1	1	0	0	0	1
FLCC291	ISP2	0	0	0	0	0	1	0	0	1	0	0	1	0	0	0	0	0	0	0	1	0	0	0	0	0	1	1	0	0	0	0	0	0	0	0	0	0	0	1	1	0	0	0	1
FLCC336	GA	0	0	0	0	0	0	0	0	0	0	0	1	0	0	0	0	0	0	1	1	0	0	0	0	0	1	0	0	0	0	0	0	0	0	0	0	0	0	1	1	0	0	0	1
FLCC336	ISP2	0	0	0	0	0	1	0	0	1	0	0	1	0	0	0	0	0	0	1	1	0	0	0	0	0	1	1	0	0	0	1	0	0	0	0	0	0	0	1	1	0	0	0	1
FLCC365	GA	0	0	0	0	0	0	0	0	1	0	0	1	0	0	0	0	0	0	1	1	0	0	0	0	0	1	1	0	0	0	0	0	0	0	0	0	0	0	1	1	0	0	0	1
FLCC365	ISP2	0	0	0	0	0	1	0	0	1	0	0	1	0	0	0	0	0	0	1	1	0	0	0	1	0	1	0	0	0	1	0	0	0	0	0	0	0	0	1	1	0	0	1	0
FLCC378	GA	NA	NA	NA	NA	NA	NA	NA	NA	NA	NA	NA	NA	NA	NA	NA	NA	NA	NA	NA	NA	NA	NA	NA	NA	NA	NA	NA	NA	NA	NA	NA	NA	NA	NA	NA	NA	NA	NA	NA	NA	NA	NA	NA	NA
FLCC378	ISP2	0	0	0	0	0	0	0	0	0	0	0	0	0	0	0	0	0	0	0	0	0	0	0	0	0	0	0	0	0	0	0	0	0	0	0	0	0	0	0	0	0	0	0	0
FLCC382	GA	0	0	0	0	0	0	0	0	0	0	0	0	0	0	0	0	0	0	0	0	0	0	0	0	0	0	0	0	0	0	0	0	0	0	0	0	0	1	0	0	0	0	0	0
FLCC382	ISP2	0	0	0	0	0	0	0	0	0	0	0	0	0	0	0	0	0	0	0	0	0	0	0	0	0	0	0	0	0	0	0	0	0	0	0	0	0	0	0	0	0	0	0	0
FLCC400	GA	0	0	0	0	1	0	0	0	1	0	0	0	0	0	0	1	0	0	0	0	0	0	0	1	0	0	1	0	0	0	0	0	0	0	0	0	1	0	1	0	0	1	0	0
FLCC400	ISP2	NA	NA	NA	NA	NA	NA	NA	NA	NA	NA	NA	NA	NA	NA	NA	NA	NA	NA	NA	NA	NA	NA	NA	NA	NA	NA	NA	NA	NA	NA	NA	NA	NA	NA	NA	NA	NA	NA	NA	NA	NA	NA	NA	NA
FLCC424	GA	NA	NA	NA	NA	NA	NA	NA	NA	NA	NA	NA	NA	NA	NA	NA	NA	NA	NA	NA	NA	NA	NA	NA	NA	NA	NA	NA	NA	NA	NA	NA	NA	NA	NA	NA	NA	NA	NA	NA	NA	NA	NA	NA	NA
FLCC424	ISP2	0	0	1	0	0	1	0	0	1	0	0	1	0	0	0	0	0	0	0	1	0	0	0	0	0	0	1	0	0	0	1	0	0	0	0	0	0	0	1	1	0	0	0	1
FLCC425	GA	0	0	0	0	0	0	0	0	0	0	0	0	0	0	0	0	0	0	0	0	0	0	0	0	0	0	0	0	0	0	0	0	0	0	0	0	0	0	0	0	0	0	0	0
FLCC425	ISP2	0	0	0	0	0	0	0	0	0	0	0	0	0	0	0	0	0	0	0	0	0	0	0	0	0	0	0	0	0	0	0	0	0	0	0	0	0	0	0	0	0	0	0	0
FLCC45	GA	NA	NA	NA	NA	NA	NA	NA	NA	NA	NA	NA	NA	NA	NA	NA	NA	NA	NA	NA	NA	NA	NA	NA	NA	NA	NA	NA	NA	NA	NA	NA	NA	NA	NA	NA	NA	NA	NA	NA	NA	NA	NA	NA	NA
FLCC45	ISP2	0	0	0	0	0	0	0	0	1	0	0	0	0	0	0	0	0	0	0	0	0	0	0	0	0	0	0	0	0	0	0	0	0	1	0	0	0	0	0	0	0	0	0	0
FLCC483	GA	NA	NA	NA	NA	NA	NA	NA	NA	NA	NA	NA	NA	NA	NA	NA	NA	NA	NA	NA	NA	NA	NA	NA	NA	NA	NA	NA	NA	NA	NA	NA	NA	NA	NA	NA	NA	NA	NA	NA	NA	NA	NA	NA	NA
FLCC483	ISP2	0	0	0	0	0	0	0	0	0	0	0	0	0	0	0	0	0	0	0	0	0	0	0	0	0	0	0	0	0	0	0	0	0	0	0	0	0	0	0	0	0	0	0	0
FLCC487	GA	NA	NA	NA	NA	NA	NA	NA	NA	NA	NA	NA	NA	NA	NA	NA	NA	NA	NA	NA	NA	NA	NA	NA	NA	NA	NA	NA	NA	NA	NA	NA	NA	NA	NA	NA	NA	NA	NA	NA	NA	NA	NA	NA	NA
FLCC487	ISP2	0	1	0	0	0	0	0	0	0	0	1	0	0	0	0	0	0	0	0	0	0	0	0	1	0	0	0	0	0	0	0	0	0	0	0	1	0	0	0	0	0	0	0	0
FLCC517	GA	0	0	0	0	0	0	0	0	0	0	1	0	0	0	0	1	0	0	0	0	0	0	0	1	0	0	0	0	0	0	0	0	0	0	0	1	1	0	1	0	0	0	0	0
FLCC517	ISP2	0	0	0	0	0	0	0	0	0	0	1	0	0	0	0	1	0	0	0	0	0	0	0	1	0	0	0	0	0	0	0	0	0	0	0	1	0	0	1	0	1	1	0	1
FLCC536	GA	NA	NA	NA	NA	NA	NA	NA	NA	NA	NA	NA	NA	NA	NA	NA	NA	NA	NA	NA	NA	NA	NA	NA	NA	NA	NA	NA	NA	NA	NA	NA	NA	NA	NA	NA	NA	NA	NA	NA	NA	NA	NA	NA	NA
FLCC536	ISP2	0	0	1	0	0	1	0	0	1	0	0	1	0	0	0	0	0	0	0	1	0	0	0	0	1	1	0	1	0	1	1	0	0	0	0	0	0	0	1	1	0	0	0	1
FLCC572	GA	NA	NA	NA	NA	NA	NA	NA	NA	NA	NA	NA	NA	NA	NA	NA	NA	NA	NA	NA	NA	NA	NA	NA	NA	NA	NA	NA	NA	NA	NA	NA	NA	NA	NA	NA	NA	NA	NA	NA	NA	NA	NA	NA	NA
FLCC572	ISP2	0	0	0	0	0	0	0	0	0	0	0	0	0	0	0	0	0	0	0	0	0	0	0	0	0	0	0	0	0	0	0	0	0	0	0	0	0	0	0	0	0	0	0	0
FLCC580	GA	NA	NA	NA	NA	NA	NA	NA	NA	NA	NA	NA	NA	NA	NA	NA	NA	NA	NA	NA	NA	NA	NA	NA	NA	NA	NA	NA	NA	NA	NA	NA	NA	NA	NA	NA	NA	NA	NA	NA	NA	NA	NA	NA	NA
FLCC580	ISP2	0	0	0	0	0	1	0	0	1	0	0	1	0	0	0	0	0	0	0	0	0	0	0	0	1	1	1	0	0	0	1	0	0	0	0	1	0	0	1	1	0	0	0	1
FLCC594	GA	0	0	0	0	0	0	0	0	1	0	0	1	0	0	0	0	0	0	1	0	1	0	1	0	0	1	0	0	1	0	0	0	0	0	0	1	0	0	1	1	0	0	0	1
FLCC594	ISP2	0	0	0	0	1	0	0	0	1	0	0	1	0	0	0	0	0	0	1	1	0	0	0	0	0	1	1	0	1	0	0	0	0	0	0	1	0	0	1	1	0	0	0	1
FLCC627	GA	NA	NA	NA	NA	NA	NA	NA	NA	NA	NA	NA	NA	NA	NA	NA	NA	NA	NA	NA	NA	NA	NA	NA	NA	NA	NA	NA	NA	NA	NA	NA	NA	NA	NA	NA	NA	NA	NA	NA	NA	NA	NA	NA	NA
FLCC627	ISP2	0	0	0	0	0	0	0	0	0	0	0	1	0	0	0	0	0	0	0	1	0	0	0	0	0	1	0	0	0	0	0	0	0	0	0	0	0	0	1	1	0	0	0	0
FLCC649	GA	NA	NA	NA	NA	NA	NA	NA	NA	NA	NA	NA	NA	NA	NA	NA	NA	NA	NA	NA	NA	NA	NA	NA	NA	NA	NA	NA	NA	NA	NA	NA	NA	NA	NA	NA	NA	NA	NA	NA	NA	NA	NA	NA	NA
FLCC649	ISP2	0	0	0	0	0	0	0	0	0	0	0	0	0	0	0	0	0	0	1	1	0	0	0	0	0	1	0	0	0	1	0	0	0	0	0	0	0	0	1	1	0	0	0	1
FLCC662	GA	NA	NA	NA	NA	NA	NA	NA	NA	NA	NA	NA	NA	NA	NA	NA	NA	NA	NA	NA	NA	NA	NA	NA	NA	NA	NA	NA	NA	NA	NA	NA	NA	NA	NA	NA	NA	NA	NA	NA	NA	NA	NA	NA	NA
FLCC662	ISP2	0	0	0	0	0	0	0	0	0	0	0	0	0	0	0	0	0	0	0	0	0	0	0	0	0	0	0	0	0	0	0	0	0	0	0	0	0	0	0	0	0	0	0	0
FLCC663	GA	NA	NA	NA	NA	NA	NA	NA	NA	NA	NA	NA	NA	NA	NA	NA	NA	NA	NA	NA	NA	NA	NA	NA	NA	NA	NA	NA	NA	NA	NA	NA	NA	NA	NA	NA	NA	NA	NA	NA	NA	NA	NA	NA	NA
FLCC663	ISP2	0	0	0	0	0	0	0	0	0	0	0	0	0	0	0	0	0	0	0	0	0	0	0	0	0	0	0	0	0	0	0	0	0	0	0	0	0	0	0	0	0	0	0	0
FLCC678	GA	NA	NA	NA	NA	NA	NA	NA	NA	NA	NA	NA	NA	NA	NA	NA	NA	NA	NA	NA	NA	NA	NA	NA	NA	NA	NA	NA	NA	NA	NA	NA	NA	NA	NA	NA	NA	NA	NA	NA	NA	NA	NA	NA	NA
FLCC678	ISP2	0	0	0	0	0	0	0	0	0	0	1	0	0	0	0	0	0	0	0	0	0	0	0	1	0	0	0	0	0	0	0	0	0	0	0	1	0	0	0	0	1	0	0	0
FLCC682	GA	NA	NA	NA	NA	NA	NA	NA	NA	NA	NA	NA	NA	NA	NA	NA	NA	NA	NA	NA	NA	NA	NA	NA	NA	NA	NA	NA	NA	NA	NA	NA	NA	NA	NA	NA	NA	NA	NA	NA	NA	NA	NA	NA	NA
FLCC682	ISP2	0	0	0	0	0	0	0	0	1	0	0	1	0	0	0	0	0	0	1	1	0	0	0	0	0	1	0	0	0	1	0	0	0	0	0	0	0	0	1	1	0	0	0	1
FLCC712	GA	NA	NA	NA	NA	NA	NA	NA	NA	NA	NA	NA	NA	NA	NA	NA	NA	NA	NA	NA	NA	NA	NA	NA	NA	NA	NA	NA	NA	NA	NA	NA	NA	NA	NA	NA	NA	NA	NA	NA	NA	NA	NA	NA	NA
FLCC712	ISP2	0	0	0	0	0	0	0	0	0	0	0	1	0	0	0	0	0	0	1	1	0	0	0	0	0	1	1	0	0	0	0	0	0	0	0	0	0	0	1	1	0	0	0	1
ms115	GA	0	0	0	0	0	0	0	0	1	0	0	0	0	0	0	0	0	0	1	0	0	0	0	0	0	1	0	0	0	1	0	0	1	0	0	0	0	0	1	1	0	0	0	1
ms115	ISP2	0	0	0	0	0	1	0	0	1	0	0	1	0	0	1	0	0	0	1	1	0	0	0	0	0	1	0	0	0	1	0	0	0	0	0	0	0	0	1	1	0	0	0	1
or3	GA	0	0	0	0	0	1	1	0	1	0	1	1	0	0	0	0	0	0	1	1	0	0	0	0	0	1	0	0	0	1	0	0	0	0	0	0	0	1	1	1	0	0	0	1
or3	ISP2	0	0	0	0	0	0	1	0	1	0	1	1	0	0	0	0	0	0	1	1	0	0	0	0	0	1	0	0	0	1	0	0	0	0	0	0	0	0	1	1	0	0	0	1
t99	GA	0	0	0	0	0	1	0	0	1	0	0	1	0	0	0	0	0	0	1	1	0	0	0	0	0	1	0	0	0	1	0	0	0	0	0	0	0	0	1	1	0	0	0	1
t99	ISP2	0	1	0	0	0	1	0	0	1	0	1	1	0	0	1	0	0	0	1	1	0	0	1	0	0	1	0	0	0	1	0	0	0	0	0	0	0	0	1	1	0	0	0	1
wa1063	GA	0	0	0	0	0	0	0	0	1	0	0	1	0	0	0	0	0	0	1	1	0	0	0	0	0	0	1	0	0	1	0	0	0	0	0	0	0	0	1	1	0	0	0	1
wa1063	ISP2	0	0	0	0	0	1	0	0	1	0	0	1	0	0	0	0	0	0	1	1	0	0	1	0	0	1	0	0	0	1	0	0	0	0	0	0	0	0	1	1	0	0	0	1
**Sample Presence**		4	5	2	1	11	18	2	2	43	1	12	47	1	1	4	3	2	2	41	36	2	2	7	8	3	44	18	1	11	17	8	1	2	2	1	9	2	5	54	50	2	3	6	44

		746.7	750.0	753.3	756.7	760.0	763.3	766.7	770.0	773.3	776.7	783.3	786.7	790.0	803.2	806.5	809.7	812.9	819.4	822.6	825.8	829.0	832.3	835.5	854.8	861.3	871.0	880.6	893.5	915.6	918.8	925.0	928.1	940.6	943.8	946.9	953.1	981.3	996.9	1000.0	
Strain	Media	31.3*	31.4	31.5	31.6	31.7	31.8	31.9	32.0	32.1	32.2	32.4	32.5	32.6	33.0	33.1	33.2	33.3	33.5	33.6	33.7	33.8	33.9	34.0	34.6	34.8	35.1	35.4	35.8	36.5	36.6	36.8	36.9	37.3	37.4	37.5	37.7	38.6	39.1	39.2	40.0
B16927	GA	0	0	0	0	0	0	0	1	1	0	0	0	1	0	0	1	0	0	0	0	0	0	0	0	0	0	0	0	0	0	0	0	0	0	0	0	0	0	0	0
B16927	ISP2	0	0	0	0	0	0	0	1	0	0	0	0	1	1	0	1	0	0	0	0	0	0	0	0	0	0	0	0	0	0	0	1	0	1	0	0	0	0	0	0
B2000	GA	0	0	0	0	0	0	0	0	0	0	0	0	0	0	0	0	0	0	0	0	0	0	0	0	0	0	0	0	0	0	0	0	0	0	0	0	0	0	1	0
B2000	ISP2	0	0	0	0	0	0	0	1	0	0	0	0	0	0	0	0	0	0	0	0	0	0	0	0	0	0	0	0	0	0	0	0	0	0	0	0	0	0	1	0
B2682	GA	0	0	0	0	0	0	0	1	0	0	0	0	0	0	0	0	0	0	0	0	0	0	0	0	0	0	0	0	0	0	0	0	0	0	0	0	0	0	0	0
B2682	ISP2	0	1	0	0	0	0	0	1	0	0	0	0	0	0	0	0	0	1	1	0	0	0	1	0	0	0	0	0	0	0	0	0	0	0	0	0	0	0	0	0
B2690	GA	0	0	0	0	0	0	0	1	0	0	0	0	0	0	0	0	0	0	0	0	0	0	0	0	0	0	0	0	0	0	0	0	0	0	0	0	0	0	0	1
B2690	ISP2	0	0	0	0	0	0	0	1	0	0	0	0	0	0	0	0	0	0	0	0	0	0	0	0	0	0	0	0	0	0	0	0	0	0	0	0	0	0	0	1
B2808	GA	0	1	0	0	0	0	0	1	0	0	0	0	0	0	0	0	0	0	1	0	0	0	0	1	0	0	0	0	0	0	0	0	0	0	0	0	0	0	0	0
B2808	ISP2	0	1	0	0	0	0	0	1	0	0	0	0	0	0	0	0	0	1	0	0	0	0	0	1	1	0	0	0	0	0	0	1	0	0	1	0	0	0	0	0
CLCC1251	GA	0	0	0	0	0	0	0	0	0	0	0	0	0	0	0	0	0	0	0	0	0	0	0	0	0	0	0	0	0	0	0	0	0	0	0	0	0	0	0	0
CLCC1251	ISP2	0	0	0	0	0	0	0	1	0	0	0	0	0	0	0	0	0	0	0	0	0	0	0	0	0	0	0	0	0	0	0	0	0	0	0	0	0	0	0	0
CLCC1393	GA	0	0	0	0	0	0	0	0	0	0	0	0	0	0	0	0	0	0	0	0	0	0	0	0	0	0	0	0	0	0	0	0	0	0	0	0	0	0	0	0
CLCC1393	ISP2	0	0	0	0	0	0	0	0	0	0	0	0	0	0	0	0	0	0	0	0	0	0	0	0	0	0	0	0	0	0	0	0	0	0	0	0	0	0	0	0
CLCC1488	GA	0	0	0	0	0	0	0	0	0	1	0	0	0	0	0	0	0	0	0	0	0	0	0	1	0	0	0	0	0	0	0	0	0	0	0	1	0	0	0	0
CLCC1488	ISP2	NA	NA	NA	NA	NA	NA	NA	NA	NA	NA	NA	NA	NA	NA	NA	NA	NA	NA	NA	NA	NA	NA	NA	NA	NA	NA	NA	NA	NA	NA	NA	NA	NA	NA	NA	NA	NA	NA	NA	NA
CLCC371A	GA	0	0	0	0	0	0	0	1	0	0	0	0	0	0	0	0	0	0	1	0	0	0	0	0	0	0	0	0	0	0	0	0	0	0	0	0	0	0	0	0
CLCC371A	ISP2	0	0	0	0	0	0	0	1	0	0	0	0	0	0	0	0	0	0	0	0	0	0	0	0	0	0	0	0	0	0	0	0	0	0	0	0	0	0	0	0
CLCC460	GA	0	0	0	0	0	0	1	0	0	0	0	0	0	0	0	0	0	0	0	0	0	0	0	0	0	0	0	1	0	0	0	0	0	0	0	0	0	0	0	0
CLCC460	ISP2	0	1	0	0	0	0	1	0	0	0	0	0	0	0	0	0	0	0	0	0	0	0	0	0	0	0	0	0	0	0	0	1	0	0	0	0	0	0	0	0
CLCC501	GA	0	0	0	0	0	0	0	0	0	0	0	0	0	0	0	0	0	0	0	0	0	0	0	0	0	0	0	0	0	0	0	0	0	0	0	0	0	0	0	0
CLCC501	ISP2	0	0	0	0	0	0	0	1	0	0	0	0	0	0	0	0	0	0	0	0	0	0	0	0	0	0	0	0	0	0	0	0	0	0	0	0	0	0	0	0
CLCC567	GA	NA	NA	NA	NA	NA	NA	NA	NA	NA	NA	NA	NA	NA	NA	NA	NA	NA	NA	NA	NA	NA	NA	NA	NA	NA	NA	NA	NA	NA	NA	NA	NA	NA	NA	NA	NA	NA	NA	NA	NA
CLCC567	ISP2	0	0	0	0	0	0	0	1	0	0	0	0	0	0	0	0	1	0	0	0	0	0	1	0	1	0	0	0	0	0	1	0	0	0	0	0	0	0	0	0
CLCC686	GA	0	0	0	0	0	0	1	1	0	0	0	0	0	0	0	0	0	0	0	0	0	0	0	1	0	0	0	0	0	0	0	0	0	0	0	0	0	0	0	0
CLCC686	ISP2	0	0	0	0	0	0	0	1	0	0	0	0	0	0	0	0	0	0	0	0	0	0	0	0	0	0	0	0	0	0	0	0	0	0	0	0	0	0	0	0
CLCC811	GA	0	0	0	0	0	1	0	1	0	0	0	0	0	0	0	0	0	0	0	0	0	0	0	1	0	0	0	0	0	0	0	0	0	0	0	0	0	0	0	0
CLCC811	ISP2	0	0	0	0	0	1	0	1	0	0	0	0	0	0	0	0	0	0	0	0	0	0	0	1	0	0	0	0	0	0	0	0	0	0	0	0	0	0	0	0
CLCC866	GA	0	0	0	0	0	0	0	0	0	0	0	0	0	0	0	0	0	0	0	0	0	0	0	0	0	0	0	0	0	0	0	0	0	0	0	0	0	0	0	0
CLCC866	ISP2	0	0	0	0	0	0	0	1	0	0	0	0	0	0	0	0	0	0	0	0	0	0	0	0	0	0	0	0	0	0	0	0	0	0	0	0	0	0	0	0
FLCC167	GA	NA	NA	NA	NA	NA	NA	NA	NA	NA	NA	NA	NA	NA	NA	NA	NA	NA	NA	NA	NA	NA	NA	NA	NA	NA	NA	NA	NA	NA	NA	NA	NA	NA	NA	NA	NA	NA	NA	NA	NA
FLCC167	ISP2	0	0	0	1	0	0	1	0	0	1	0	0	0	1	0	0	0	0	0	0	0	0	0	1	1	0	0	0	0	0	0	1	0	0	0	0	0	0	0	0
FLCC191	GA	NA	NA	NA	NA	NA	NA	NA	NA	NA	NA	NA	NA	NA	NA	NA	NA	NA	NA	NA	NA	NA	NA	NA	NA	NA	NA	NA	NA	NA	NA	NA	NA	NA	NA	NA	NA	NA	NA	NA	NA
FLCC191	ISP2	0	1	0	0	0	0	0	1	0	0	0	0	0	0	0	0	0	0	0	0	0	0	1	0	1	0	0	0	0	0	0	1	0	0	0	0	0	0	0	0
FLCC204	GA	0	0	0	0	1	0	0	1	0	0	0	1	0	0	0	0	0	0	0	0	0	0	0	0	0	0	0	0	0	0	0	0	0	0	0	0	0	0	0	0
FLCC204	ISP2	0	1	0	0	1	0	0	1	1	0	0	1	0	0	0	0	0	1	0	1	0	0	1	0	1	0	0	0	1	0	1	0	0	0	0	0	0	0	0	0
FLCC211	GA	0	0	0	0	0	0	0	0	0	0	0	0	0	0	0	0	0	0	0	0	0	0	0	0	0	0	0	0	0	0	0	0	0	0	0	0	0	0	0	0
FLCC211	ISP2	0	0	0	0	0	0	0	0	0	0	0	0	0	0	0	0	0	0	0	0	0	0	0	0	0	0	0	0	0	0	0	0	0	0	0	0	0	0	0	0
FLCC251	GA	0	0	0	0	0	0	0	0	0	0	0	0	0	0	0	0	0	0	0	0	0	0	0	0	0	0	0	0	0	0	0	0	0	0	0	0	0	0	0	0
FLCC251	ISP2	0	0	0	0	0	0	0	0	0	0	0	0	0	0	0	0	0	0	0	0	0	0	0	0	0	0	0	0	0	0	0	0	0	0	0	0	0	0	0	0
FLCC270	GA	0	0	0	0	0	0	0	0	0	0	0	0	0	0	0	0	0	0	0	0	0	0	0	0	0	0	0	0	0	0	0	0	0	0	0	0	0	0	0	0
FLCC270	ISP2	0	0	0	0	0	0	0	0	0	0	0	0	0	0	0	0	0	0	0	0	0	0	0	0	0	0	0	0	0	0	0	0	0	0	0	0	0	0	0	0
FLCC291	GA	0	0	0	0	1	0	0	0	0	0	0	0	0	0	0	0	0	0	0	0	0	0	0	0	0	0	0	0	0	0	0	0	0	0	0	0	0	0	0	0
FLCC291	ISP2	0	0	0	0	0	0	0	0	0	0	0	0	0	0	0	0	0	0	0	0	0	0	0	0	0	0	0	0	0	0	0	0	0	0	0	0	0	0	0	0
FLCC336	GA	0	0	0	0	0	0	0	0	0	0	0	0	0	0	0	0	0	0	0	0	0	0	0	0	0	0	0	0	0	0	0	1	0	0	0	0	0	0	0	0
FLCC336	ISP2	0	0	0	0	0	0	0	0	0	0	0	0	0	0	0	0	0	0	0	0	0	0	0	0	0	0	0	0	0	0	0	0	0	0	0	0	0	0	0	0
FLCC365	GA	0	0	0	0	0	1	0	0	0	0	0	0	0	0	0	0	0	0	0	0	0	0	0	0	0	0	0	0	0	0	0	0	0	0	0	0	0	0	0	0
FLCC365	ISP2	0	0	0	1	0	0	1	1	0	0	0	0	0	0	0	0	0	1	0	1	0	0	0	0	0	0	0	0	1	0	0	0	0	0	0	0	0	0	0	0
FLCC378	GA	NA	NA	NA	NA	NA	NA	NA	NA	NA	NA	NA	NA	NA	NA	NA	NA	NA	NA	NA	NA	NA	NA	NA	NA	NA	NA	NA	NA	NA	NA	NA	NA	NA	NA	NA	NA	NA	NA	NA	NA
FLCC378	ISP2	0	0	0	0	0	0	0	0	0	0	0	0	0	0	0	0	0	0	0	0	0	0	0	0	0	0	0	0	0	0	0	0	0	0	0	0	0	0	0	0
FLCC382	GA	0	0	0	0	0	0	0	0	0	0	0	0	0	0	0	0	0	0	0	0	0	0	0	0	0	0	0	0	0	0	0	0	0	0	0	0	0	0	0	0
FLCC382	ISP2	0	0	0	0	0	0	0	0	0	0	0	0	0	0	0	0	0	0	0	0	0	0	0	0	0	0	0	0	0	0	0	0	0	0	0	0	0	0	0	0
FLCC400	GA	0	0	0	0	0	0	1	0	0	1	0	0	0	0	0	0	0	0	0	0	0	0	0	1	0	1	0	0	0	0	0	1	1	0	0	1	0	1	0	0
FLCC400	ISP2	NA	NA	NA	NA	NA	NA	NA	NA	NA	NA	NA	NA	NA	NA	NA	NA	NA	NA	NA	NA	NA	NA	NA	NA	NA	NA	NA	NA	NA	NA	NA	NA	NA	NA	NA	NA	NA	NA	NA	NA
FLCC424	GA	NA	NA	NA	NA	NA	NA	NA	NA	NA	NA	NA	NA	NA	NA	NA	NA	NA	NA	NA	NA	NA	NA	NA	NA	NA	NA	NA	NA	NA	NA	NA	NA	NA	NA	NA	NA	NA	NA	NA	NA
FLCC424	ISP2	0	0	0	0	0	0	0	0	0	0	0	0	0	0	0	0	0	0	0	0	0	0	0	0	0	0	0	0	0	0	0	1	0	0	0	0	0	0	0	0
FLCC425	GA	0	0	0	0	0	0	0	0	0	0	0	0	0	0	0	0	0	0	0	0	0	0	0	0	0	0	0	0	0	0	0	0	0	0	0	0	0	0	0	0
FLCC425	ISP2	0	0	0	0	0	0	0	1	0	0	0	0	0	0	0	0	0	0	0	0	0	0	0	0	0	0	0	0	0	0	0	0	0	0	0	0	0	0	0	0
FLCC45	GA	NA	NA	NA	NA	NA	NA	NA	NA	NA	NA	NA	NA	NA	NA	NA	NA	NA	NA	NA	NA	NA	NA	NA	NA	NA	NA	NA	NA	NA	NA	NA	NA	NA	NA	NA	NA	NA	NA	NA	NA
FLCC45	ISP2	0	0	0	0	0	0	0	1	0	0	0	0	0	0	0	0	0	0	0	0	0	0	0	0	0	0	0	0	0	0	0	0	0	0	0	0	0	0	0	0
FLCC483	GA	NA	NA	NA	NA	NA	NA	NA	NA	NA	NA	NA	NA	NA	NA	NA	NA	NA	NA	NA	NA	NA	NA	NA	NA	NA	NA	NA	NA	NA	NA	NA	NA	NA	NA	NA	NA	NA	NA	NA	NA
FLCC483	ISP2	0	0	0	0	0	0	0	1	0	0	0	0	0	0	0	0	0	0	0	0	0	0	0	0	0	0	0	0	0	0	0	0	0	0	0	0	0	0	0	0
FLCC487	GA	NA	NA	NA	NA	NA	NA	NA	NA	NA	NA	NA	NA	NA	NA	NA	NA	NA	NA	NA	NA	NA	NA	NA	NA	NA	NA	NA	NA	NA	NA	NA	NA	NA	NA	NA	NA	NA	NA	NA	NA
FLCC487	ISP2	0	0	0	0	0	0	1	0	0	1	0	0	0	1	0	0	0	0	0	0	0	0	0	1	0	0	0	0	0	0	0	0	0	0	0	1	0	0	0	0
FLCC517	GA	0	0	0	0	0	1	0	0	0	1	0	0	0	1	0	0	0	0	0	0	0	0	0	1	1	0	0	0	0	0	0	1	0	0	0	0	0	0	0	0
FLCC517	ISP2	0	0	0	0	0	1	1	0	0	1	0	0	0	1	0	0	0	0	0	0	0	1	0	1	1	0	0	0	0	0	1	0	0	0	0	0	0	0	0	0
FLCC536	GA	NA	NA	NA	NA	NA	NA	NA	NA	NA	NA	NA	NA	NA	NA	NA	NA	NA	NA	NA	NA	NA	NA	NA	NA	NA	NA	NA	NA	NA	NA	NA	NA	NA	NA	NA	NA	NA	NA	NA	NA
FLCC536	ISP2	0	0	0	0	1	0	1	0	0	0	0	0	0	0	0	0	0	0	0	0	0	0	0	0	0	0	0	0	0	0	0	0	0	0	0	0	0	0	0	0
FLCC572	GA	NA	NA	NA	NA	NA	NA	NA	NA	NA	NA	NA	NA	NA	NA	NA	NA	NA	NA	NA	NA	NA	NA	NA	NA	NA	NA	NA	NA	NA	NA	NA	NA	NA	NA	NA	NA	NA	NA	NA	NA
FLCC572	ISP2	0	0	0	0	0	0	0	1	0	0	0	0	0	0	0	0	0	0	0	0	0	0	0	0	0	0	0	0	0	0	0	0	0	0	0	0	0	0	0	0
FLCC580	GA	NA	NA	NA	NA	NA	NA	NA	NA	NA	NA	NA	NA	NA	NA	NA	NA	NA	NA	NA	NA	NA	NA	NA	NA	NA	NA	NA	NA	NA	NA	NA	NA	NA	NA	NA	NA	NA	NA	NA	NA
FLCC580	ISP2	0	0	0	0	0	1	0	1	0	0	0	0	0	0	0	0	0	0	0	0	0	0	0	0	1	0	0	0	0	0	0	0	0	0	0	0	0	0	0	0
FLCC594	GA	0	0	0	0	0	0	0	1	0	0	0	0	0	0	0	0	0	0	0	0	0	0	0	1	0	0	0	0	0	0	0	0	0	0	0	0	0	0	0	0
FLCC594	ISP2	1	1	0	0	0	1	0	1	0	0	0	0	0	0	0	0	0	0	0	0	0	0	0	1	0	0	0	0	0	0	0	0	0	0	0	0	0	0	0	0
FLCC627	GA	NA	NA	NA	NA	NA	NA	NA	NA	NA	NA	NA	NA	NA	NA	NA	NA	NA	NA	NA	NA	NA	NA	NA	NA	NA	NA	NA	NA	NA	NA	NA	NA	NA	NA	NA	NA	NA	NA	NA	NA
FLCC627	ISP2	0	0	0	0	0	0	0	0	0	0	0	0	0	0	0	0	0	0	0	0	0	0	0	0	0	0	0	0	0	0	0	0	0	0	0	0	0	0	0	0
FLCC649	GA	NA	NA	NA	NA	NA	NA	NA	NA	NA	NA	NA	NA	NA	NA	NA	NA	NA	NA	NA	NA	NA	NA	NA	NA	NA	NA	NA	NA	NA	NA	NA	NA	NA	NA	NA	NA	NA	NA	NA	NA
FLCC649	ISP2	0	0	0	0	0	0	0	0	0	0	0	0	0	0	0	0	0	0	0	0	0	0	0	0	0	0	0	0	0	0	0	0	0	0	0	0	0	0	0	0
FLCC662	GA	NA	NA	NA	NA	NA	NA	NA	NA	NA	NA	NA	NA	NA	NA	NA	NA	NA	NA	NA	NA	NA	NA	NA	NA	NA	NA	NA	NA	NA	NA	NA	NA	NA	NA	NA	NA	NA	NA	NA	NA
FLCC662	ISP2	0	0	0	0	0	0	0	0	0	0	0	0	0	0	0	0	0	0	0	0	0	0	0	0	0	0	0	0	0	0	0	0	0	0	0	0	0	0	0	0
FLCC663	GA	NA	NA	NA	NA	NA	NA	NA	NA	NA	NA	NA	NA	NA	NA	NA	NA	NA	NA	NA	NA	NA	NA	NA	NA	NA	NA	NA	NA	NA	NA	NA	NA	NA	NA	NA	NA	NA	NA	NA	NA
FLCC663	ISP2	0	0	0	0	0	0	0	0	0	0	0	0	0	0	0	0	0	0	0	0	0	0	0	0	0	0	0	0	0	0	0	0	0	0	0	0	0	0	0	0
FLCC678	GA	NA	NA	NA	NA	NA	NA	NA	NA	NA	NA	NA	NA	NA	NA	NA	NA	NA	NA	NA	NA	NA	NA	NA	NA	NA	NA	NA	NA	NA	NA	NA	NA	NA	NA	NA	NA	NA	NA	NA	NA
FLCC678	ISP2	0	0	0	0	0	0	1	0	0	0	0	0	0	1	0	0	0	0	0	0	0	0	0	1	0	0	0	0	0	0	0	0	0	0	0	0	0	0	0	0
FLCC682	GA	NA	NA	NA	NA	NA	NA	NA	NA	NA	NA	NA	NA	NA	NA	NA	NA	NA	NA	NA	NA	NA	NA	NA	NA	NA	NA	NA	NA	NA	NA	NA	NA	NA	NA	NA	NA	NA	NA	NA	NA
FLCC682	ISP2	0	0	0	0	1	0	0	1	0	0	0	0	0	0	0	0	0	0	0	0	0	0	0	0	0	0	0	0	0	0	0	0	0	0	0	0	0	0	0	0
FLCC712	GA	NA	NA	NA	NA	NA	NA	NA	NA	NA	NA	NA	NA	NA	NA	NA	NA	NA	NA	NA	NA	NA	NA	NA	NA	NA	NA	NA	NA	NA	NA	NA	NA	NA	NA	NA	NA	NA	NA	NA	NA
FLCC712	ISP2	0	1	0	0	1	0	0	1	0	0	0	0	0	0	0	0	1	0	0	0	0	0	1	0	0	0	0	0	0	0	1	0	0	0	0	0	0	0	0	0
ms115	GA	0	0	0	0	0	0	1	1	0	0	1	0	0	0	0	0	0	1	0	0	0	0	0	1	0	0	0	0	0	1	0	0	0	0	1	0	0	0	0	0
ms115	ISP2	0	0	0	0	0	0	0	1	0	0	0	0	0	0	0	0	0	1	0	0	0	0	0	0	0	0	0	0	0	1	0	0	0	0	0	0	0	0	0	0
or3	GA	0	0	1	0	0	0	1	1	0	0	0	0	0	0	1	0	0	1	1	1	0	0	0	0	0	0	1	0	0	1	0	0	0	0	0	0	1	0	0	0
or3	ISP2	0	0	1	0	0	0	1	1	0	0	0	0	0	0	0	0	0	0	1	1	0	0	0	0	0	0	0	0	0	0	0	0	0	0	0	0	1	0	1	0
t99	GA	0	0	0	0	0	0	1	1	0	0	0	0	0	0	0	0	0	0	0	0	0	0	0	0	0	0	0	0	0	1	0	0	0	0	0	0	0	0	0	0
t99	ISP2	1	0	0	0	0	0	1	1	0	0	0	0	0	0	0	0	0	1	1	0	0	0	1	0	0	0	0	0	0	1	0	0	0	0	0	0	0	0	0	0
wa1063	GA	0	0	0	0	0	0	1	0	0	0	0	0	0	0	0	0	0	0	0	0	1	0	0	0	0	0	1	0	0	0	0	0	0	0	0	0	1	0	1	0
wa1063	ISP2	0	0	0	0	0	0	1	1	0	0	0	0	0	0	0	0	0	1	0	1	0	0	0	0	0	0	0	0	0	0	0	0	0	0	0	0	1	0	0	0
**Sample Presence**		2	8	2	2	6	7	17	39	2	6	1	2	2	6	1	2	2	9	6	5	1	1	6	15	8	1	2	1	2	5	4	9	1	1	2	3	4	1	4	2
